# A scoping review on hyposalivation associated with systemic conditions: the role of physical stimulation in the treatment approaches

**DOI:** 10.1186/s12903-023-03192-8

**Published:** 2023-07-21

**Authors:** Jéssica Luiza de Mendonça Albuquerque Melo, Camila Pinho e Souza Coelho, Fernanda de Paula e Silva Nunes, Debora Heller, Daniela Corrêa Grisi, Maria do Carmo Machado Guimarães, Naile Dame-Teixeira

**Affiliations:** 1 Department of Dentistry, School of Health Sciences, University of Brasilia, Brasília, Brazil; 2 Post Graduate Program in Dentistry, Cruzeiro Do Sul University, São Paulo, Brazil; 3 Experimental Research, Hospital Israelita Albert Einstein, São Paulo, Brazil; 4 Department of Periodontology, UT Health San Antonio, San Antonio, TX USA; 5 Oral Biology Division, School of Dentistry, University of Leeds, Leeds, UK

**Keywords:** Hyposalivation, Autoimmune disease, Salivary stimulation, Transcutaneous electrical nerve stimulation, Low power laser, Acupuncture

## Abstract

**Background:**

Several systemic conditions can result in distinct degrees of salivary gland damage and consequent hypofunction. The development of successful management schemes is highly challenging due to the complexity of saliva. This study aimed to systematically map the literature on the physical stimulation of salivary glands for hyposalivation management and the response of individuals according to different systemic conditions causing salivary impairment.

**Methods:**

A systematic search in the literature was performed. Two reviewers independently selected clinical trials, randomized or not, that used physical stimulation to treat hyposalivation caused by systemic conditions. Studies evaluating healthy subjects without hyposalivation were included as controls. Single-arm clinical studies or case series were also included for protocol mapping (PRISMA extension for scoping reviews).

**Results:**

Out of 24 included studies, 10 evaluated healthy subjects, from which 9 tested transcutaneous electrical nerve stimulation (TENS) and 1 tested acupuncture and electroacupuncture. Fourteen studies evaluated individuals with hyposalivation: 6 applied TENS, 6 applied low-level laser therapy (LLLT), and 2 applied acupuncture, carried out in post-chemotherapy, medication use, postmenopausal women, hemodialysis patients, smokers, diabetics, Sjögren's syndrome (SS). All showed increased salivation after treatment, except for two LLLT studies in individuals with SS.

**Conclusions:**

Among the different patient groups, individuals with Sjögren's syndrome (SS) exhibited the poorest responses, while those with medication-induced hyposalivation demonstrated the most favorable treatment outcomes, independently of the management strategy for saliva stimulation. It means that physical stimulation of salivary glands holds promise as an alternative for managing hyposalivation in cases of reversible gland damage. However, to make informed decisions in current practice, it is necessary to conduct new well-designed randomized clinical trials with appropriate methodologies.

**Supplementary Information:**

The online version contains supplementary material available at 10.1186/s12903-023-03192-8.

## Background

Saliva is essential for maintaining oral health [1, 2]. This abundant biological fluid contains a wide mineral content, inflammatory biomarkers, proteins, peptides, and nucleic acids, including non-coding RNA. Very recently, saliva has been employed for identifying the presence of miRNAs as diagnostic and prognostic indicators of oral potentially malignant disorders [3] and Sjögren's syndrome (SS) [4], as well as offers sensitivity and specificity for SARS-CoV-2 detection [5].The reduction in salivary flow (hyposalivation) and the dry mouth sensation (xerostomia) compromise health, significantly affecting social and emotional aspects of life [6–9]. The hyposalivation plays a crucial role in the development of oral diseases, such as caries, periodontitis, candidiasis, inflammation, and atrophic changes stand out of the oral mucosa, ulcerations and opportunistic infections [1, 8, 10, 11].

A wide range of systemic diseases and conditions are capable of affecting salivary secretion, such as diabetes [12], Sjögren's syndrome (SS) [13], hypertension, hypothyroidism, as well as clinical conditions that require the administration of anticholinergic drugs [14]. These drugs cause a reversible effect on the salivary glands by competing with muscarinic receptors in the salivary glands [14]. As examples, drugs of recurrent use with antihistamine, antidepressant, antihypertensive, antiparkinsonian, and anxiolytic effects are anticholinergic agents most commonly associated with adverse effects on the salivary glands [14, 15]. On the other hand, prolonged hyperglycemia in diabetes can lead to increased urine production and consequent dehydration, insufficiency of parasympathetic stimulation, or a change in the membranes of the salivary glands [12]. Finally, SS can cause hyposalivation by damaging the glandular parenchyma in an autoimmune response [4].

A significant challenge faced by many clinicians is the recovery of the salivary flow in these conditions. Among the alternatives for the management of hyposalivation, pilocarpine and cevimeline are cholinergic agonist drugs that have been widely used for the chemical stimulation of salivary secretion [16]. However, such strategies often result in systemic adverse effects that may include nausea, fever, diarrhoea, and sweating, in addition to presenting contraindications for some patients [17, 18]. As alternatives to these drugs, several researchers suggested physical stimulations of salivary flow, such as using low-level laser treatment (LLLT) [18], acupuncture [19] and transcutaneous electrical nerve stimulation (TENS) [20, 21]. These alternatives are more conservative, less invasive, less costly and with no or few adverse effects [17]. Low-level laser and acupuncture treatment could improve salivary flow by similar mechanisms: they increase microcirculation through the release of sensory neuropeptides, and increase tissue oxygenation and metabolism [18, 19, 22]. Additionally, the laser seems to contribute to glandular tissue repair [18]. As for TENS, the mechanism of action in the parotid gland is still unclear, but might have an effect by stimulating the auriculotemporal nerve [17, 20, 21, 23]. However, there is limited information in the literature regarding these approaches [16, 24–27]. Some reviews were detected, but they either tested single therapies [27], or include patients with only one associated condition, such as SS [26]. Furthermore, by including only randomized clinical trials or studies on irradiated patients that usually have severe and irreversible glands damage, other reviews restricted an assessment of the available protocols [24, 25, 28]. Different systemic conditions result in different degrees of damage of the salivary glands, as well as distinct etiologies. Notwithstanding, clinical protocols considering these particularities to manage hyposalivation are of utmost importance. This scoping review aimed to map all protocols available for physical stimuli of the salivary gland for hyposalivation managementin systemically compromised individuals. As a secondary objective, we aimed to undertand whether individuals with different conditions respond differently to treatments.

## Methods

### Study design

This reviews followed the PRISMA (Preferred Reporting Items for Systematic Reviews and Meta-Analyses) Extension for Scoping Reviews checklist [29]. The research question was: “What are the current protocols using physical stimulation of salivary glands for the management of hyposalivation caused by systemic conditions or diseases?’’.

### Search strategy

A wide-open systematic search of the literature was conducted in electronic databases (MEDLINE/PubMed, Cochrane Library, Scopus, Livivo, Embase, Web of Science) as well as the gray literature (Google Scholar and ProQuest). Keywords and general controlled vocabularies (MeSH terms) were selected, without the restriction of language, year or type of publication. Terms included ''hyposalivation'', ''autoimmune disease'', ''salivary stimulation'', ''transcutaneous electrical nerve stimulation'', ''low-level light therapy'', ''acupuncture'', ' 'treatment''. Duplicate references were removed by the EndNoteWeb reference manager (Clarivate Analytics, Mumbai) and then manually (Online Resource; Appendix Table 1).

### Eligibility criteria

Studies were included if they satisfied all the following criteria: randomized clinical trial, non-randomized clinical trial, single-arm clinical trial and case reports, that used physical methods to treat hyposalivation caused by systemic conditions or healthy. Studies were excluded if: 1) the target population included irradiated patients, prosthesis wearers, pediatric subjects, or had unknown cause for hyposalivation, 2) Only chewing gum or other non-physical method, or presented confounding bias such as sialogogues and salivary substitutes, 3) had clinical results without quantitative analysis of the salivary flow, 4) were not original research, 5) were conference abstracts or study protocols, or 6) were written in a non-Latin alphabet, without the possibility of translation by tools such as Google Translator.

### Selection of the manuscripts

Two reviewers (J.L.M.A.M. and C.P.S.C.) independently screened the eligibility of all identified titles and abstracts using the Rayyan QCRI® open access tool (Qatar Computer Research Institute, Qatar). The same reviewers also evaluated full-text articles for inclusion using the same eligibility criteria. Disagreements between the reviewers at this stage were discussed with a third reviewer (F.P.S.N.) until a consensus was reached and the conflict was resolved. The third reviewer is an expert in the field and has over fifteen years of experience. The reference lists of the selected articles were manually analyzed to identify other potential studies to inclusion.

### Data extraction and synthesis

**Fig. 1:**
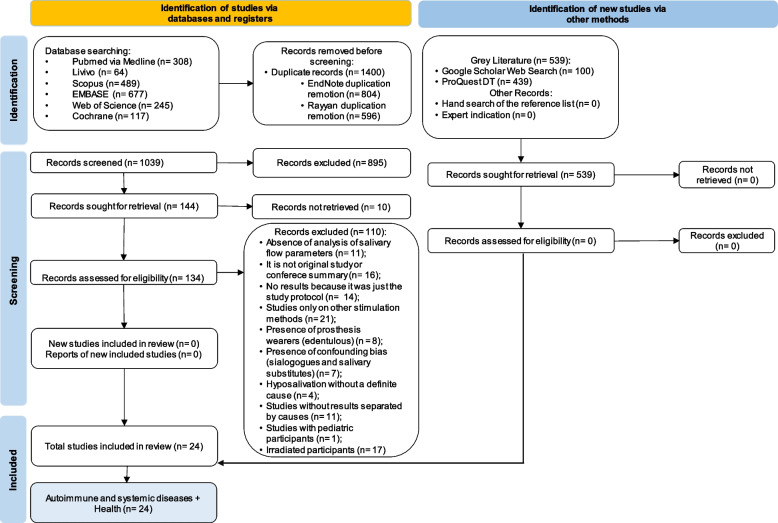
Flow diagram for selection of studies according to the PRISMA (Preferred Reporting Items for Systematic Reviews and Meta-Analyses) guidelines for scoping reviews

Data extraction was performed by independent reviewers (J.L.M.A.M. and C.P.S.C) in a table designed to this study, including the following information: author (year), country, study design, the age range of individuals, cause of hyposalivation, diagnosis of hyposalivation, type of treatment, salivary flow prior to treatment, salivary flow after treatment. All extracted data were verified by a third reviewer (F.P.S.N.). The data was summarized and described according to the general characteristics of the studies, type of treatment and condition. Figure 1 presents a PRISMA flow diagram outlining the study selection process. The *GraphPad Prism* software (version 9.5.0 for Mac) was used for data synthesis and graph production.

## Results

### Studies characteristics

**Fig. 2:**
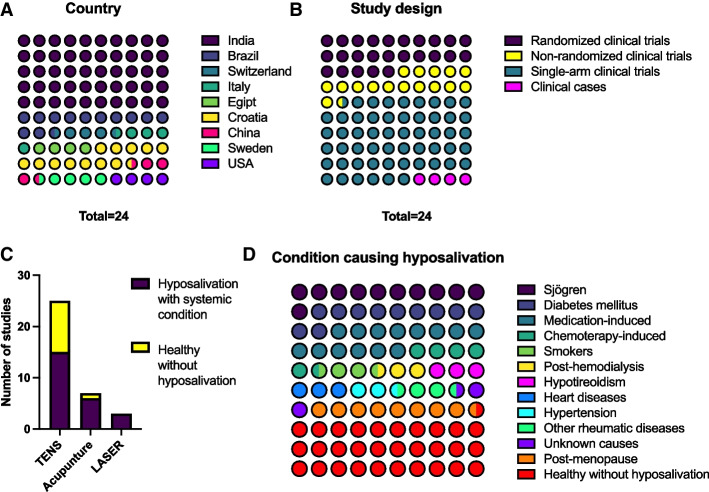
The distribution of the 24 included studies by (**A**) country, (**B**) according to studies design and (**C**) according to the physical methods of salivary stimulation and the target population, or (**D**) according to the systemic condition associated with hyposalivation or for healthy subjects without hyposalivation. The color scale indicates the number of studies in the “parts of whole graphs”, as shown in their right

Out of the 1900 titles recovered in the search, 144 were included in the first phase and 24 were included after full-text reading. Six were pilot studies or had preliminary results. Among the included articles, 14 applied physical stimuli to salivary glands for managing hyposalivation caused by diseases or systemic conditions and 10 applied the methods of salivary glands stimulation in healthy people (Fig. 2c). Those studies were included as controls, to understand how a normal gland would react to these treatments. The Online Resources, Appendix table 2 depicted the excluded studies. A total number of 1262 subjects were evaluated in 12 studies from India, 3 from Brazil, 1 from Switzerland, 1 from Italy, 1 from Egypt, 1 from Croatia, 1 from China, 1 from Sweden and 1 from the United States (Fig. 2a).

Only 6 studies were randomized clinical trials (RCTs), with sample sizes ranging from 9 to 33 participants. Their control groups consisted of superficial acupuncture, laser application with the device turned off, laser with different power and wavelength, hyperboloid (masticatory device in silicone), as well as groups without any treatment. Four studies were non-randomized clinical trials, with sample ranging from 3 to 38 participants. Their parallel groups corresponded to the application of other methods (such as spraying water), different protocols (such as different TENS frequencies), use of TENS in healthy individuals or in the ones without hyposalivation. However, most included studies (*n* = 13) were single-arm clinical trials, and 1 was a case report (Fig. 2b).

Among the RCTs, 2 studies evaluated participants with medication-induced hyposalivation, 2 with SS, 1 after chemotherapy, and 1 evaluated participants with different conditions: heart disease, hypertension, post-menopause, using medication, unknown cause, hypothyroidism, rheumatic diseases, SS and others. The other studies evaluated participants with hyposalivation caused by diabetes, tobacco use, post-chemotherapy treatment, medication use, post-hemodialysis, post-menopause and SS (Table 1) (Online Resources, Appendix table 3) (Fig. 2d).

**Table 1:** Detailed protocol from therapies used for salivary stimulation of salivary glands to management of hyposalivation caused by systemic diseases or conditions. For the characteristics and main results of each study, see Appendix Table 3

Author, country, year, type of study	Condition causing hyposalivation	Physical method used for salivary glands stimulation	Protocol (number of sections in bold)
Aggarwal et al. India 2015 SACT [30]	Healthy subjects without hyposalivation	TENS	Electrodes bilaterally on the skin overlying the parotid gland region Frequency 100 Hz and pulse duration 100-150 µs After a 2-min break, the TENS unit was activated and the amplitude was increased to a maximum tolerated level for 5 min **Single application**
Amaral et al. Brazil 2012 RCT [31]	Chemothrapy-induced	TENS	G1 = Pulse frequency of 50 Hz and pulse duration of 250 microseconds 3 sessions weekly for 30 min each Electrodes in the 3 regions of the face that corresponded to the major salivary glands G2 = TENS + Hyperboloid; G3 = Without salivary stimulation; G4 = Balanced masticatory exercises after meals using a hyperboloid (mechanical sialogogue), for 10 min, 4 × a day **Patients underwent therapy from day 7 before hematopoietic stem cell transplantation (D-7) to day 30 after transplantation (D30)**
Bhasin et al. India 2015 SACT [32]	Healthy subjects without hyposalivation	TENS	Electrodes bilaterally on the skin overlying the parotid gland region The device used operates with a frequency of 0.1 to 500 Hz. Amplitude was increased to a maximum tolerable level for the patient for 5 min **Single application**
Blom et al. Switzerland 1992 RCT [19]	SS, Medication-induced, unknown cause, hypothyroidism, heart disease, hypertension, rheumatic diseases, postmenopausa	Acupuncture	G1 = 6 to 8 local and distal points and 2 to 4 auricular points, with insertions at depths between 0.5 and 2.0 cm and approximately 1 to 3 mm into the ear G2 = intraoperative needles dermal (superficial acupuncture) G1 and G2 = **2x/week, 20 min, for 6 weeks. Interval of 7 to 10 days and protocol repetition for another 6 weeks**
Brzak et al. Croatia 2017 RCT [33]	Medication-induced	Laser	G1 = Power of 35mW and wavelength of 830 nm. 5.2 Hz pulse repetition rate G2 = Power of 30mW and wavelength of 685 nm. 5.2 Hz pulse repetition rate Approximately 14.4 J per session in G1 and G2 6 irradiation points. Each application with different duration depending on the gland and laser wavelength **The treatment lasted 10 consecutive days for G1 and G2**
Cafaro et al. Italy 2014 RCT [34]	SS	Laser acupuncture	G1 = Light in the red visible spectrum (650 nm), power of 5 mW, 120 s per acupuncture point, total dose of 0.6 J 1x/week for 5 weeks (5 sessions)6 acupuncture points stimulated bilaterally G2 = G1 protocol, but without emitting radiation
Chandra et al. India 2022 NRCT [35]	Diabetes, chemothrapy-induced, smokers, healthy subjects without hyposalivation	TENS	Electrodes bilaterally on the skin overlying the parotid gland region Frequency between 20–50 Hz, intensity according to the patient's tolerance **2x/week every 15 days, for 1 month (4 times in total)**
Dabic ´ et al. Croatia 2016 RCT [22, 36]	Medication-induced	Laser	G1 = Wavelength of 830 nm, power of 35 mW, frequency of 5.2 Hz, alternating mode (on: 800 ms, off: 1 ms) and dose of 1.60 J/cm^2^ Extraoral and intraoral irradiation points (does not inform quantity) Each session lasted 20 min, 10 min per side of the face (total of 120 s of irradiation per session) **The laser was repeated every day, except on weekends, for 14 days (10 sessions)** G2 = Same protocol, but with the device turned off
Dabic ´ et al. Croatia 2016 NRCT [22, 36]	Medication-induced	Acupuncture	G1 = 5 acupuncture points on both ears for 30 min Participants received press needles in one ear to be used until the second session (1 week later). The remaining three sessions were every seven days (5 sessions) G2 = Water in a spray bottle (0.5 L) was given to participants to use ad libitum for 2 weeks. The patients did not know that it was pure water
Dawidson et al. Sweden 1997 SACT [37]	Healthy subjects without hyposalivation	Acupuncture and electroacupuncture	Acupuncture at 6 points bilaterally The same patients received low-frequency electrical stimulation (2 Hz) evoked with a Multiple Electronic Acupuntoscope. The needles at points St6 and Li4 were connected to the electropulse bilaterally, and the electrical current was adjusted between 2–4 mA. Electrical stimulation was applied for 20 min **Single application**
Dyasnoor et al. India 2017 SACT [21]	Diabetes	TENS	Electrodes bilaterally on the skin overlying the parotid gland region Pulse rate fixed at 50 Hz and intensity according to patient tolerance **Single application**
Fidelix et al. Brazil 2018 RCT [38]	SS	Laser	G1 = Wavelength 808 nm, 100 mW, and in continuous wave mode 2x/week for 6 weeks (**12 sessions**) 12 extraoral irradiation points and 2 intraoral irradiation points 4 J per stitch for 40 s. Total energy dose per session: 56 J G2 = G1 protocol, but without emitting radiation
Hargitai et al. United States of America 2005 SACT [39]	Healthy subjects without hyposalivation	TENS	Electrodes bilaterally on the skin overlying the parotid gland region The pulse rate was fixed at 50 Hz, the pulse duration was fixed at 250 ms and the unit was in normal mode. Reach the maximum intensity tolerable by the patient. Application for 5 min Single application
Konidena et al. India 2016 NRCT [40]	Postmenopausa	TENS	Electrodes bilaterally on the skin overlying the parotid gland region 50 Hz frequency, 220 V, 0–100 mA at 1 k load, biphasic waveform, available in pulsed/continuous form and in 2 intensities The TENS unit was activated in continuous mode, with maximum intensity tolerated by the patient for 15 min (40 patients supported intensity II); **Single application**
Nimma et al. India 2012 SACT [41]	Healthy subjects without hyposalivation	TENS	Electrodes bilaterally on the skin overlying the parotid gland region The pulse rate was fixed at 50 Hz and the amplitude was the maximum tolerated by the patient. Application for 5 min **Method applied on 2 consecutive days**
Pandey et al. India 2019 SACT [42]	Healthy subjects without hyposalivation	TENS	**Day 1:** Citric acid stimulation for all subjects. Rinse with 5 mL of citric acid solution (25%) for 15 s **2nd day:** Stimulation with TENS for all individuals. Electrodes bilaterally on the skin overlying the parotid gland region. The pulse rate was fixed at 50 Hz and the amplitude was the maximum tolerated by the patient. Application for 5 min
Pattipati et al. India 2013 SACT [43]	Healthy subjects without hyposalivation	TENS	Electrodes bilaterally on the skin overlying the parotid gland region 50 Hz pulse rate, unit in normal mode **Single application.** No information regarding TENS application time
Ramesh et al. India 2021 SACT [44]	Healthy subjects without hyposalivation	TENS	Electrodes bilaterally on the skin overlying the parotid gland region The pulse rate was fixed at 50 Hz and the amplitude was the maximum tolerated by the patient. Application for 5 min **Single application**
Shetawy et al. Egypt 2021 SACT [45]	Diabetes	Laser	905 nm wavelength, 100mW power, 2 J energy per point and 28 J dose per session 2x/week for 6 weeks (**total of 12 sessions**) 14 points of extraoral irradiation The application time was 30 s per point (total duration of 420 s)
Simões et al. Brazil 2009 CR [46]	SS	Laser	G1 = Wavelength of 780 nm, power of 15mW, in continuous wave mode, dose of 3.8 J/cm^2^ **For 8 months, treatment was given 3x/week for 4 weeks, followed by a 1-week break, and then treatment was resumed** 10 s per point. 30 extraoral irradiation points and 6 intraoral points
Singh et al. India 2015 SACT [47]	Healthy subjects without hyposalivation	TENS	Electrodes bilaterally on the skin overlying the parotid gland region The amplitude was the maximum tolerated by the patient. Application for 5 min. Rate and pulse were not reported **Single application**
Smriti et al. India 2014 SACT [23]	Medication-induced, postmenopausa, diabetes	TENS	Electrodes bilaterally on the skin overlying the parotid gland region Pulse rate of 50 Hz, duration of 250 microseconds, unit in normal mode up to the maximum intensity tolerated by the patient **Single application**
Vilas et al. India 2009 SACT [48]	Healthy subjects without hyposalivation	TENS	Electrodes bilaterally on the skin overlying the parotid gland region The pulse rate was fixed at 50 Hz and the amplitude was the maximum tolerated by the patient. Application for 5 min **Single application**
Yang et al. China 2022 NRCT [49]	Hemodialysis	TENS on acupuncture points	G1 = Frequency of 50 Hz and a pulse duration of 250 μs. Electrodes were placed on 2 acupuncture points bilaterally and the stimulation lasted for 20 min G2 = Same treatment as G1, but with a frequency of 2 Hz and a pulse of 50 μs

*RCT* Randomized clinical trial, *NRCT* Non-randomized clinical trial, *SACT* Single-arm clinical trial, *CR* Case report, *TENS* Transcutaneous electric nerve stimulation, *SS* Sjogren syndrome

### Management strategies for salivary stimulation

Table 1 describes the protocols used for each study. Treatments included low-level light therapy (LLLT), transcutaneous electrical nerve stimulation (TENS), acupuncture and electroacupuncture (Fig. 2c). However, direct comparison (studies with at least two parallel arms) was performed solely in 4 studies. LLLT was tested for SS patients [34, 38, 46] and for medication-induced hyposalivation [22, 33]. Few studies applied acupuncture in healthy individuals [37], medication-induced hyposalivation [36], or in various systemic conditions at the same study [19]. Electroacupuncture was tested for healthy individuals without hyposalivation [37]. TENS was investigated in 16 studies, analyzing healthy individuals [30, 32, 35, 39, 41–44, 47, 48], diabetics [21, 23, 35], postmenopausal women [23], patients on chemotherapy [31, 35], and on hemodialysis [49].

Increase of salivary flow by treatment and different conditions causing hyposalivation.

The groups compared in the RCTs were LLLT vs. placebo in patients on medication, LLLT vs. placebo in SS, and TENS vs. placebo in patients after chemotherapy, totaling 171 individuals. The randomization was not fully described in most clinical trials [22, 31, 34, 38]. Calibration of examiners and any blinding process was described in a few studies [34, 38], and none followed the CONSORT [50].

In the study conducted in patients with different systemic conditions, the acupuncture had improved salivary flow rates during and after treatment, and the results persisted after 12 months. Likewise, the group that received superficial (intradermal) acupuncture, considered as a placebo, had a satisfactory response in salivary flow as well. However, these improvement was not sustained after the end of the protocol [19].

**Fig. 3:**
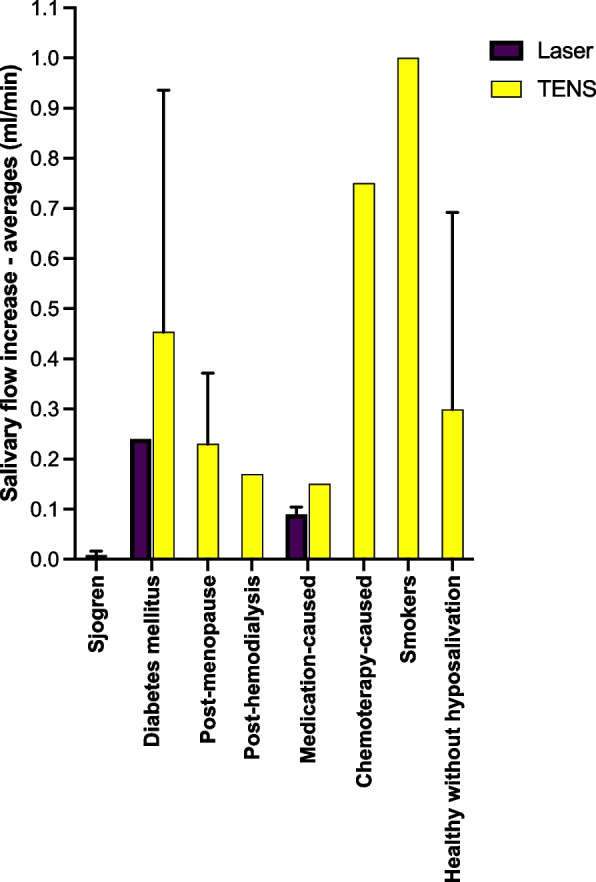
Average increase in salivary flow according to the associated systemic condition and the applied physical method

A study evaluating SS patients who were treated with LLLT showed an increase in salivary flow at the end of the protocol, greater than the placebo group, reporting a stability until the 3rd month of follow-up and a reduction from the 3^rd^ to the 6^th^month [34]. However, divergent results were observed in another similar study (no difference in the salivary flow using laser vs. placebo) [38]. Nevertheless, the LLLT presented positive results for treating patients with drug-associated hyposalivation [22, 33]. This was confirmed by calculating cross-study averages of the salivary increase, suggesting that SS is the worst condition to recover the function of salivary glands. Meanwhile, other conditions such as smoking can present more reversible effect in the salivary glands (Fig. 3).

Most non-randomized clinical trials tested the effect of TENS in subjects with hyposalivation caused by conditions as varied as diabetes, smokers, post-chemotherapy, healthy, post-menopausal, and post-hemodialysis. For these studies, all tested subjects had increased salivary flow, and among the ones with hyposalivation, diabetics had a better response to treatment [35]. From the 13 single-arm trials showing positive effects of salivary glands physical stimuli, 12 applied TENS, from which 9 were samples of healthy individuals. Eight of these studies were carried out in India, with sample numbers ranging from 50 to 130 individuals. Other single-arm trials tested TENS in diabetic patients [21, 23] and in samples of postmenopausal and drug-associated hyposalivation [23]. When the TENS was compared with mechanical salivary stimulation (by chewing of a silicone hyperboloid after meals), before and after transplantation of hematopoietic stem cells, it was observed a preventive reduction of salivary flow and a tendency to increase salivation [31].

Considering the cause-related hyposalivation described in all types of the included studies, better results in salivary flow increase and maintenance were observed when hyposalivation was related to the use of medications. Only 2 studies did not result in increased salivary flow, both treating hyposalivation in individuals with SS [38, 46].

### Hyposalivation management protocols

Regarding the protocols presented by the studies, there were a wide variation in the time of salivary gland stimulation used by each method, in the follow-up time, and the stimulation parameters used (such as TENS frequency, wavelength, power and total energy delivered by the laser and acupuncture points). Table 1 and Online Resources, Appendix table 3 contain all protocols applied for the different causes of hyposalivation.

Different studies used varying protocols for acupuncture. The longest study lasted for 12 weeks with two sessions per week, using multiple points on the head, hands, legs, and auricular points [19]. Another study applied acupuncture to auricular points in five sessions [36]. Yet another study used only six body points in a single session [37]. These differences demonstrate the variety of protocols used in acupuncture studies.

The application points for laser treatment were similar across studies, both extraoral and intraoral. However, the wavelength, power, and total energy varied significantly between studies. Some studies even used different wavelengths in separate groups, indicating that different effects can be expected with different laser protocols. Most studies used different wavelengths, except for two studies that agreed on using 830 nm in their test groups.

As for TENS, the studies that evaluated its effects were mostly with frequency adjustment at 50 Hz and variable pulse duration (which was not described in details for all cases). As for TENS, most studies evaluating its effects used a frequency of 50 Hz and variable pulse duration (which was not described in details for all cases). The electrodes were generally placed bilaterally in the parotid gland region for a single application lasting 5 min. One study applied TENS to acupuncture points for 20 min. Most studies reported adjusting the TENS intensity based on the patient's tolerance level. [35].

Only 4 studies monitored sialometry in addition to that performed immediately after treatment application [19, 33, 34, 49]. In general, the individuals did not report any type of complaint or discomfort during the application of the physical methods.

## Discussion

This scoping review aimed to map the studies evaluating available methods of physical stimulation of salivary glands for the management of hyposalivation in systemically compromised individuals, as well as in healthy controls. We also qualitatively analysed the outcomes of the different therapeutic approaches for hyposalivation. Studies included in this review associated physical approaches to treat hyposalivation caused by several conditions. Studies that represent high-quality evidence—RCTs—to assess the efficacy of interventions are limited. There are a very small number of studies with a parallel-group, with or without treatment [19, 22, 31, 33, 34, 38] which would allow a more comprehensive comparison between the interventions. Nevertheless, the decision for including of all forms of clinical studies is justified to ensure the literature mapping regarding available protocols.

The studies included in this analysis exhibit certain limitations, primarily of a methodological nature. Few RCTs provided sufficient details regarding randomization, blinding, and examiner calibration. It is worth considering that the use of CONSORT guidelines has been associated with improved quality in reporting RCTs [51]. The absence of studies utilizing such a tool may suggest a potential low quality of evidence for the reported findings. Furthermore, the outcome of the study may be influenced by knowledge of the allocation, which characterizes the observation bias in the case of non-blinded studies. Obtaining evidence of efficacy is also hampered by methodological problems such as choosing a control in studies. As an example, for the acupuncture method, 1 study used superficial acupuncture [19] and 1 study used water as a comparative group [36]. As superficial acupuncture and the use of spray water may have specific effects of their own, evidence derived from such studies may underestimate the benefits of acupuncture. Studies using TENS present very significant sample sizes, with consistent results of increased salivary flow after stimulation. However, the lack of control groups, either a placebo or a gold standard, as well as long term evaluations, make it impossible to confirm its efficacy. It is crucial to emphasize that the evaluation of study quality did not influence the inclusion process, as the primary objective of the review was to comprehensively provide an overview of the existing research regardless of their quality assessment.

Hyposalivation can be manifested together or separately from xerostomia, but it is important to understand that they are two distinct conditions associated with the state of dry mouth [52]. The reduction in salivary flow serves as an objective indication of hyposalivation, although there is limited strong evidence regarding the specific volume of saliva deemed healthy. Conversely, xerostomia, the most prevalent salivary disorder, is reported by over 50% of individuals and refers to the persistent subjective sensation of a dry mouth experienced by the patient [2, 11]. Many studies assessed the impact of treatments on the quality of life associated with xerostomia, taking into account that this condition is common and may or may not be accompanied by hyposalivation. The perception of dry mouth is recognized as a significant variable that can greatly affect the quality of life related to oral health. Therefore, understanding whether treatments that increase salivary flow also alleviate xerostomia is relevant for improving the overall well-being of individuals experiencing these oral health challenges [6, 9]. While some studies found no statistically significant differences or slight flow increase without reaching normal levels [53], these treatments could have some effect in the management of xerostomia improving patients’ quality of life, which should be better investigated. For instance, it was reported less than 10% of salivary flow increased (from 0.033 to 0.036 ml/min), but the subjects reported improvement in lubrication and mouth moisture during laser therapy [46]. It is important to point out that we did not collected information of xerostomia recover after these treatments, and the outcome was the increase in the saliva volume.

Hyposalivation caused by the use of medication was the most repeated condition within the studies, being cited by 5 of them. Two of these studies used acupuncture as a treatment method [19, 36], 2 used laser [22, 33] and 1 applied TENS [23]. Only two studies described the category of drugs administered [19, 23]. Considering that the drugs do not cause physical damage to salivary gland cells and that the receptor binding process is simple and reversible, the better results obtained by these patients can be justified [14]. Nevertheless, included studies do not report whether the individuals were under continuous use or if they stopped using such drugs during the follow-up, which is a factor difficulting this analysis.

On the other hand, unfavorable responses were associated with SS [38, 46]. This can be explained by the complex mechanism through which SS affects the glandular tissue, initially affecting the glandular epithelium [54] and gradually progressing to fibrosis and complete destruction of the glandular parenchyma [55], although the precise causes and mechanisms underlying SS are not completely comprehended. One hypothesis proposes a dysregulation of the immune system when certain individuals are exposed to particular environmental factors like viruses [4]. Thus, any modality of physical stimulus seems to have little or no effect due to the intense and irreversible degeneration of the glands [39].

In the present review, we decided to include post-chemotherapy patients, although irradiated subjects were excluded, due to such different mechanisms that ionizing radiation damages the salivary glands generating particular outcomes for the use of physical methods in these patients [28]. While radiotherapy acts by destroying the DNA of cells potentially sensitive to certain doses of ionizing radiation [2], chemotherapy involves the administration of cytotoxic drugs, by different routes, including the oral route, which will circulate through the bloodstream and target cells in high dose of cell division. Anyway, a single article included hemotherapy patients [3] in the scope review is a clinical trial with 33 patients who received allogeneic hematopoietic stem cell transplantation. Chemotherapy was used prior to this transplant in these individuals to basically avoid graft rejection. In this case, the patients suffered from bone marrow aplasia, acute myeloid leukemia, acute lymphocyte leukemia, Hodgkin's lymphoma, and mantle cell lymphoma.

None of the included studies directly cited disadvantages or adverse effects (such as pain or discomfort) experienced by participants during and after salivary stimulation treatments. However, when observing the number of sessions of application of the methods (every week and, sometimes, more than once a week for months), we can infer some disadvantages related to these treatments such as the displacement to the health center, periods of treatment, position during the sialometry and application of the method and, often, the need to miss work. While many studies mention that patients did not report discomfort during the application of the techniques, there is a lack of clear evidence regarding potential adverse effects. Therefore, we recommend that future clinical trials investigating salivary stimulation using physical methods delve deeper into exploring and addressing the logistical disadvantages associated with established protocols, as we previously discussed in our publication by Coelho et al. [28].

This scoping review has certain limitations due to the relatively low number of studies available and the wide diversity in methodological approaches. This heterogeneity among the applied protocols, the scarcity of studies for specific diseases and different treatment methods, the absence of long-term follow-up, small sample sizes, and the fact that more than half of the studies did not incorporate parallel groups all restricted our ability to thoroughly evaluate the efficacy of treatments and protocols. These limitations highlight the need for further research with larger sample sizes and standardized methodologies to draw more conclusive findings.

## Conclusions

Among the different patient groups, individuals with Sjögren's syndrome (SS) exhibited the poorest responses, while those with medication-induced hyposalivation demonstrated the most favorable treatment outcomes, independently of the management strategy for saliva stimulation. It means that physical stimulation of salivary glands holds promise as an alternative for managing hyposalivation in cases of reversible gland damage. However, to make informed decisions in current practice, it is necessary to conduct new well-designed randomized clinical trials with appropriate methodologies.

## Supplementary Information

**Additional file 1. **

## Data Availability

The data that support the findings of this study will be openly available in the Online Resources Supplementary Material.

## Abbreviations

TENS: Transcutaneous electrical nerve stimulation
LLLT: Low-level laser therapy
SS: Sjögren's syndrome
PRISMA: Preferred Reporting Items for Systematic Reviews and Meta-Analyses
RCT: Randomized clinical trial
NRCT: Non-randomized clinical trial
SACT: Single-arm clinical trial
CR: Case report
