# Supplementary material for: A scoping review on hyposalivation associated with systemic conditions: the role of physical stimulation in the treatment approaches
Source: BMC Oral Health. 2023 Jul 21;23:505. doi: 10.1186/s12903-023-03192-8 (PMC10360219; doi:10.1186/s12903-023-03192-8)
Supplement: Supplementary file 1 — Additional file 1. [file 12903_2023_3192_MOESM1_ESM.pdf]

A Scoping Review on Hyposalivation associated with Systemic Conditions: The Role of Physical Stimulation in the Treatment Approaches

Jéssica Luiza Mendonça Albuquerque de Melo[1], Camila Pinho e Souza Coelho[1], Fernanda de Paula e Silva Nunes[1], Debora Heller[2, 3, 4], Daniela Corrêa Grisi[1], Maria do Carmo Machado Guimarães[1], Nailê Damé-Teixeira[1]

[1]Department of Dentistry, School of Health Sciences, University of Brasilia, Brasília, Brazil [2] Post Graduate Program in Dentistry, Cruzeiro do Sul University, São Paulo, Brazil
[3] Experimental Research, Hospital Israelita Albert Einstein, São Paulo, Brazil
[4] Department of Periodontology, UT Health San Antonio, San Antonio, TX, USA

*Corresponding Authors:
Daniela Corrêa Grisi, danigrisi@gmail.com
Naile Dame-Teixeira, nailedame@unb.br

Appendix table 1. Search strategies according to each database, carried out on 09/05/2022.

DATABASE	SEARCH STRATEGIES	NUMBER OF TITLES
Pubmed via Medline	("head and neck neoplasms"[All Fields] OR "head and neck neoplasms"[MeSH Terms] OR "neoplasms head and neck"[All Fields] OR "head and neck cancer"[All Fields] OR "head neck neoplasms"[All Fields] OR "Head and Neck Neoplasm"[All Fields] OR "Cancer of Head and Neck"[All Fields] OR "head and neck cancer"[All Fields] OR "Cancer of the Head and Neck"[All Fields] OR "Head Neoplasms"[All Fields] OR "neoplasms head"[All Fields] OR "Head Neoplasm"[All Fields] OR "neoplasm head"[All Fields] OR "Neck Neoplasms"[All Fields] OR "neoplasms neck"[All Fields] OR "Neck Neoplasm"[All Fields] OR "neoplasm neck"[All Fields] OR "Cancer of Head"[All Fields] OR "Head Cancers"[All Fields] OR "Head Cancer"[All Fields] OR "cancer head"[All Fields] OR "cancers head"[All Fields] OR "Cancer of the Head"[All Fields] OR "Cancer of Neck"[All Fields] OR "Neck Cancers"[All Fields] OR "Neck Cancer"[All Fields] OR "cancer neck"[All Fields] OR "cancers neck"[All Fields] OR "Cancer of the Neck"[All Fields] OR "radiotherapy"[All Fields] OR "radiotherapy"[MeSH Terms] OR "Radiotherapies"[All Fields] OR "Radiation Therapy"[All Fields] OR "intensity-modulated radiotherapy"[All Fields] OR "Radiation Therapies"[All Fields] OR "therapies radiation"[All Fields] OR "therapy radiation"[All Fields] OR "Radiation Treatment"[All Fields] OR "Radiation Treatments"[All Fields] OR "treatment radiation"[All Fields] OR "radiotherapy targeted"[All Fields] OR "radiotherapies targeted"[All Fields] OR "Targeted Radiotherapies"[All Fields] OR "Targeted Radiotherapy"[All Fields] OR "Targeted Radiation Therapy"[All Fields] OR ("radiotherapy"[MeSH Terms] OR "radiotherapy"[All Fields] OR ("radiation"[All Fields] AND "therapies"[All Fields] AND "targeted"[All Fields])) OR "Radiation Dosage"[All Fields] OR "Iodine Radioisotopes"[MeSH Terms] OR "Iodine Radioisotopes"[All Fields] OR "Targeted Radiation Therapies"[All Fields] OR ("radiotherapy"[MeSH Terms] OR "radiotherapy"[All Fields] OR ("therapies"[All Fields] AND "targeted"[All Fields] AND "radiation"[All Fields])) OR "therapy targeted radiation"[All Fields] OR "radiation therapy targeted"[All Fields] OR "Radiation Injuries"[All Fields] OR "radiation effects"[All Fields] OR "intensity-modulated radiotherapy"[All Fields] OR "cancer complications"[All Fields] OR "Sjogren's Syndrome"[All Fields] OR "Sjogren's Syndrome"[MeSH Terms] OR "Sjogrens Syndrome"[All Fields] OR "syndrome sjogren s"[All Fields] OR "Sjogren Syndrome"[All Fields] OR "Sicca Syndrome"[All Fields] OR "syndrome sicca"[All Fields] OR "Diabetes Mellitus"[MeSH Terms] OR "Diabetes Mellitus"[All Fields] OR "Diabetes Insipidus"[All Fields] OR "diet diabetic"[All Fields] OR "Prediabetic State"[All Fields] OR "Scleredema Adultorum"[All Fields] OR "glycation end products advanced"[All Fields] OR "Glucose Intolerance"[All Fields] OR "Gastroparesis"[All Fields] OR "Antihypertensive Agents"[MeSH Terms] OR "Antihypertensive Agents"[All Fields] OR "agents antihypertensive"[All Fields] OR "Antihypertensive Agent"[All Fields] OR "agent antihypertensive"[All Fields] OR "anti hypertensive agent"[All Fields] OR "agent anti hypertensive"[All Fields] OR "anti hypertensive agent"[All Fields] OR "anti hypertensive drug"[All Fields] OR "anti hypertensive drug"[All Fields] OR "drug anti hypertensive"[All Fields] OR "Antihypertensive Drug"[All Fields] OR "drug antihypertensive"[All Fields] OR "Antihypertensives"[All Fields] OR "anti hypertensive agents"[All Fields] OR "agents anti hypertensive"[All Fields] OR "anti hypertensive agents"[All Fields] OR "anti hypertensive drugs"[All Fields] OR "anti hypertensive drugs"[All Fields] OR "drugs anti hypertensive"[All Fields] OR "anti hypertensives"[All Fields] OR "anti hypertensives"[All Fields] OR "Antihypertensive Drugs"[All Fields] OR "drugs antihypertensive"[All Fields] OR "anti hypertensive"[All Fields] OR "anti hypertensive"[All Fields] OR "Antihypertensive"[All Fields] OR "Cystic Fibrosis"[MeSH Terms] OR "Cystic Fibrosis"[All Fields] OR "fibrosis cystic"[All Fields] OR "Mucoviscidosis"[All Fields] OR "Pulmonary Cystic Fibrosis"[All Fields] OR "cystic fibrosis pulmonary"[All Fields] OR "Pancreatic Cystic Fibrosis"[All Fields] OR "cystic fibrosis pancreatic"[All Fields] OR "Fibrocystic Disease of Pancreas"[All Fields] OR "Pancreas Fibrocystic Disease"[All Fields] OR ("Cystic Fibrosis"[MeSH Terms] OR ("cystic"[All Fields] AND "fibrosis"[All Fields]) OR "Cystic Fibrosis"[All Fields] OR ("pancreas"[All Fields] AND "fibrocystic"[All Fields] AND "diseases"[All Fields])) OR "Cystic Fibrosis of Pancreas"[All Fields] OR "Cholinergic Antagonists"[MeSH Terms] OR "Cholinergic Antagonists"[All Fields] OR "antagonists cholinergic"[All Fields] OR "Cholinergic Receptor Antagonist"[All Fields] OR ("Cholinergic Antagonists"[Pharmacological Action] OR "Cholinergic Antagonists"[MeSH Terms] OR ("cholinergic"[All	308

	Fields] AND "antagonists"[All Fields]) OR "Cholinergic Antagonists"[All Fields] OR ("antagonist"[All Fields] AND "cholinergic"[All Fields] AND "receptor"[All Fields])) OR ("Cholinergic Antagonists"[Pharmacological Action] OR "Cholinergic Antagonists"[MeSH Terms] OR ("cholinergic"[All Fields] AND "antagonists"[All Fields]) OR "Cholinergic Antagonists"[All Fields] OR ("receptor"[All Fields] AND "antagonist"[All Fields] AND "cholinergic"[All Fields])) OR "cholinergic blocking agent"[All Fields] OR ("Cholinergic Antagonists"[Pharmacological Action] OR "Cholinergic Antagonists"[MeSH Terms] OR ("cholinergic"[All Fields] AND "antagonists"[All Fields]) OR "Cholinergic Antagonists"[All Fields] OR ("agent"[All Fields] AND "cholinergic"[All Fields] AND "blocking"[All Fields])) OR "cholinergic blocking agent"[All Fields] OR "Cholinolytic"[All Fields] OR "Cholinolytics"[All Fields] OR "Acetylcholine Antagonist"[All Fields] OR "antagonist acetylcholine"[All Fields] OR "Cholinergic Antagonist"[All Fields] OR "antagonist cholinergic"[All Fields] OR "Cholinergic Receptor Antagonists"[All Fields] OR ("Cholinergic Antagonists"[Pharmacological Action] OR "Cholinergic Antagonists"[MeSH Terms] OR ("cholinergic"[All Fields] AND "antagonists"[All Fields]) OR "Cholinergic Antagonists"[All Fields] OR ("antagonists"[All Fields] AND "cholinergic"[All Fields] AND "receptor"[All Fields])) OR "receptor antagonists cholinergic"[All Fields] OR "cholinergic blocking agents"[All Fields] OR "agents cholinergic blocking"[All Fields] OR "cholinergic blocking agents"[All Fields] OR "Acetylcholine Antagonists"[All Fields] OR "antagonists acetylcholine"[All Fields] OR "Anticholinergic Agents"[All Fields] OR "agents anticholinergic"[All Fields] OR "Anticholinergic Agent"[All Fields] OR "agent anticholinergic"[All Fields] OR "Anticholinergics"[All Fields] OR "anti cholinergics"[All Fields] OR "anti cholinergics"[All Fields] OR "anti cholinergic"[All Fields] OR "anti cholinergic"[All Fields] OR "Anticholinergic"[All Fields] OR "Histamine Antagonists"[MeSH Terms] OR "Histamine Antagonists"[All Fields] OR "antagonists histamine"[All Fields] OR "Antihistamines"[All Fields] OR "Histamine Antagonist"[All Fields] OR "antagonist histamine"[All Fields] OR "Antihistamine"[All Fields] OR "Antidepressive Agents"[MeSH Terms] OR "Antidepressive Agents"[All Fields] OR "agents antidepressive"[All Fields] OR "Antidepressive Agent"[All Fields] OR "agent antidepressive"[All Fields] OR "Antidepressants"[All Fields] OR "Antidepressant"[All Fields] OR "Antidepressant Drugs"[All Fields] OR "drugs antidepressant"[All Fields] OR "Antidepressant Drug"[All Fields] OR "drug antidepressant"[All Fields] OR "Thymoleptics"[All Fields] OR "Thymoleptic"[All Fields] OR "Thymoanaleptics"[All Fields] OR "Thymoanaleptic"[All Fields] OR "Antiparkinson Agents"[MeSH Terms] OR "Antiparkinson Agents"[All Fields] OR ("Antiparkinson Agents"[Pharmacological Action] OR "Antiparkinson Agents"[MeSH Terms] OR ("antiparkinson"[All Fields] AND "agents"[All Fields]) OR "Antiparkinson Agents"[All Fields] OR ("agents"[All Fields] AND "antiparkinson"[All Fields])) OR "Antiparkinsonian Agents"[All Fields] OR ("Antiparkinson Agents"[Pharmacological Action] OR "Antiparkinson Agents"[MeSH Terms] OR ("antiparkinson"[All Fields] AND "agents"[All Fields]) OR "Antiparkinson Agents"[All Fields] OR ("agents"[All Fields] AND "antiparkinsonian"[All Fields])) OR "Antiparkinsonians"[All Fields] OR "Antiparkinson Drugs"[All Fields] OR "drugs antiparkinson"[All Fields] OR "anti anxiety agents"[MeSH Terms] OR "anti anxiety agents"[All Fields] OR "agents anti anxiety"[All Fields] OR "anti anxiety agents"[All Fields] OR "anti anxiety drug"[All Fields] OR "anti anxiety drug"[All Fields] OR "drug anti anxiety"[All Fields] OR "anti anxiety drugs"[All Fields] OR "anti anxiety drugs"[All Fields] OR "drugs anti anxiety"[All Fields] OR "Anxiolytic"[All Fields] OR "Anxiolytics"[All Fields] OR "tranquilizing agents minor"[All Fields] OR ("anti anxiety agents"[Pharmacological Action] OR "anti anxiety agents"[MeSH Terms] OR ("anti anxiety"[All Fields] AND "agents"[All Fields]) OR "anti anxiety agents"[All Fields] OR ("agents"[All Fields] AND "minor"[All Fields] AND "tranquilizing"[All Fields])) OR "Minor Tranquilizing Agents"[All Fields] OR ("anti anxiety agents"[Pharmacological Action] OR "anti anxiety agents"[MeSH Terms] OR ("anti anxiety"[All Fields] AND "agents"[All Fields]) OR "anti anxiety agents"[All Fields] OR ("tranquillizing"[All Fields] AND "agents"[All Fields] AND "minor"[All Fields])) OR ("anti anxiety agents"[Pharmacological Action] OR "anti anxiety agents"[MeSH Terms] OR ("anti anxiety"[All Fields] AND "agents"[All Fields]) OR "anti anxiety agents"[All Fields] OR ("agents"[All Fields] AND "minor"[All Fields] AND "tranquillizing"[All Fields])) OR ("anti anxiety agents"[Pharmacological Action] OR "anti anxiety agents"[MeSH Terms] OR ("anti anxiety"[All Fields] AND "agents"[All Fields]) OR "anti anxiety agents"[All Fields] OR ("minor"[All Fields] AND "tranquillizing"[All Fields] AND "agents"[All Fields])) OR "Anxiolytic Agent"[All Fields] OR ("anti anxiety agents"[Pharmacological Action] OR "anti anxiety agents"[MeSH Terms] OR ("anti anxiety"[All Fields] AND "agents"[All Fields]) OR "anti anxiety agents"[All Fields] OR ("agent"[All Fields] AND "Anxiolytic"[All Fields])) OR "anti anxiety agent"[All Fields] OR ("anti anxiety agents"[Pharmacological Action] OR "anti anxiety agents"[MeSH Terms] OR ("anti anxiety"[All Fields] AND "agents"[All Fields]) OR "anti anxiety agents"[All Fields] OR ("agent"[All Fields] AND "anti"[All Fields] AND "anxiety"[All Fields])) OR "anti anxiety agent"[All Fields] OR "Anxiolytic Agents"[All Fields] OR "agents anxiolytic"[All Fields] OR "anti anxiety effect"[All Fields] OR "anti anxiety effect"[All Fields] OR ("anti anxiety agents"[Pharmacological Action] OR "anti anxiety agents"[MeSH Terms] OR ("anti anxiety"[All Fields] AND "agents"[All Fields]) OR "anti anxiety agents"[All Fields] OR ("effect"[All Fields] AND "anti"[All Fields] AND "anxiety"[All Fields])) OR "Anxiolytic Effects"[All Fields] OR "effects anxiolytic"[All Fields] OR "Antianxiety Effects"[All Fields] OR ("anti anxiety agents"[Pharmacological Action] OR "anti anxiety agents"[MeSH Terms] OR ("anti anxiety"[All Fields] AND "agents"[All Fields]) OR "anti anxiety agents"[All Fields] OR ("effects"[All Fields] AND "antianxiety"[All Fields])) OR "Anxiolytic Effect"[All Fields] OR "effect anxiolytic"[All Fields] OR "anti anxiety effects"[All Fields] OR "anti anxiety effects"[All Fields] OR "effects anti anxiety"[All Fields] OR "Antianxiety Effect"[All Fields] OR "effect antianxiety"[All Fields] OR "Obesity"[MeSH Terms] OR "Obesity"[All Fields] OR "xerostomia"[All Fields] OR "xerostomia"[MeSH Terms] OR "Xerostomias"[All Fields] OR "xerostomy"[All Fields] OR "Hyposalivation"[All Fields] OR "Hyposalivations"[All Fields] OR "Asialia"[All Fields] OR "Salivary gland dysfunction"[All Fields] OR "Mouth Dryness"[All Fields] OR "dryness mouth"[All Fields] OR "oral dryness"[All Fields] OR "Salivary hypo-function"[All Fields] OR "dry mouth"[All Fields] OR "Radiation-induced xerostomia"[All Fields]) AND ("Transcutaneous Electric Nerve Stimulation"[MeSH Terms] OR "Transcutaneous Electric Nerve Stimulation"[All Fields] OR "electric stimulation transcutaneous"[All Fields] OR "stimulation	

	transcutaneous electric"[All Fields] OR "Transcutaneous Electric Stimulation"[All Fields] OR "Percutaneous Electric Nerve Stimulation"[All Fields] OR "TENS"[All Fields] OR "electrical stimulation transcutaneous"[All Fields] OR "Transcutaneous Electrical Stimulation"[All Fields] OR "Transdermal Electrostimulation"[All Fields] OR ("Transcutaneous Electric Nerve Stimulation"[MeSH Terms] OR ("transcutaneous"[All Fields] AND "electric"[All Fields] AND "nerve"[All Fields] AND "stimulation"[All Fields]) OR "Transcutaneous Electric Nerve Stimulation"[All Fields] OR ("electrostimulation"[All Fields] AND "transdermal"[All Fields])) OR "Percutaneous Electrical Nerve Stimulation"[All Fields] OR "Transcutaneous Electrical Nerve Stimulation"[All Fields] OR "Transcutaneous Nerve Stimulation"[All Fields] OR "nerve stimulation transcutaneous"[All Fields] OR "stimulation transcutaneous nerve"[All Fields] OR "Percutaneous Neuromodulation Therapy"[All Fields] OR ("Transcutaneous Electric Nerve Stimulation"[MeSH Terms] OR ("transcutaneous"[All Fields] AND "electric"[All Fields] AND "nerve"[All Fields] AND "stimulation"[All Fields]) OR "Transcutaneous Electric Nerve Stimulation"[All Fields] OR ("neuromodulation"[All Fields] AND "therapy"[All Fields] AND "percutaneous"[All Fields])) OR "Percutaneous Neuromodulation Therapies"[All Fields] OR ("Transcutaneous Electric Nerve Stimulation"[MeSH Terms] OR ("transcutaneous"[All Fields] AND "electric"[All Fields] AND "nerve"[All Fields] AND "stimulation"[All Fields]) OR "Transcutaneous Electric Nerve Stimulation"[All Fields] OR ("therapy"[All Fields] AND "percutaneous"[All Fields] AND "neuromodulation"[All Fields])) OR "Percutaneous Electrical Neuromodulation"[All Fields] OR ("Transcutaneous Electric Nerve Stimulation"[MeSH Terms] OR ("transcutaneous"[All Fields] AND "electric"[All Fields] AND "nerve"[All Fields] AND "stimulation"[All Fields]) OR "Transcutaneous Electric Nerve Stimulation"[All Fields] OR ("electrical"[All Fields] AND "neuromodulation"[All Fields] AND "percutaneous"[All Fields])) OR ("Transcutaneous Electric Nerve Stimulation"[MeSH Terms] OR ("transcutaneous"[All Fields] AND "electric"[All Fields] AND "nerve"[All Fields] AND "stimulation"[All Fields]) OR "Transcutaneous Electric Nerve Stimulation"[All Fields] OR ("electrical"[All Fields] AND "neuromodulations"[All Fields] AND "percutaneous"[All Fields])) OR ("Transcutaneous Electric Nerve Stimulation"[MeSH Terms] OR ("transcutaneous"[All Fields] AND "electric"[All Fields] AND "nerve"[All Fields] AND "stimulation"[All Fields]) OR "Transcutaneous Electric Nerve Stimulation"[All Fields] OR ("neuromodulation"[All Fields] AND "percutaneous"[All Fields] AND "electrical"[All Fields])) OR ("Transcutaneous Electric Nerve Stimulation"[MeSH Terms] OR ("transcutaneous"[All Fields] AND "electric"[All Fields] AND "nerve"[All Fields] AND "stimulation"[All Fields]) OR "Transcutaneous Electric Nerve Stimulation"[All Fields] OR ("neuromodulations"[All Fields] AND "percutaneous"[All Fields] AND "electrical"[All Fields])) OR ("Transcutaneous Electric Nerve Stimulation"[MeSH Terms] OR ("transcutaneous"[All Fields] AND "electric"[All Fields] AND "nerve"[All Fields] AND "stimulation"[All Fields]) OR "Transcutaneous Electric Nerve Stimulation"[All Fields] OR ("percutaneous"[All Fields] AND "electrical"[All Fields] AND "neuromodulations"[All Fields])) OR "Chewing Gums"[All Fields] OR "Chewing Gum"[MeSH Terms] OR "Chewing Gum"[All Fields] OR "gum chewing"[All Fields] OR "gums chewing"[All Fields] OR "Chewing"[All Fields] OR "low level light therapy"[All Fields] OR "low level light therapy"[MeSH Terms] OR ("low level light therapy"[MeSH Terms] OR ("low level"[All Fields] AND "light"[All Fields] AND "therapy"[All Fields]) OR "low level light therapy"[All Fields] OR ("light"[All Fields] AND "therapies"[All Fields] AND "low"[All Fields] AND "level"[All Fields])) OR "light therapy low level"[All Fields] OR "low level light therapy"[All Fields] OR "Low-Level Light Therapies"[All Fields] OR ("low level light therapy"[MeSH Terms] OR ("low level"[All Fields] AND "light"[All Fields] AND "therapy"[All Fields]) OR "low level light therapy"[All Fields] OR ("therapies"[All Fields] AND "low"[All Fields] AND "level"[All Fields] AND "light"[All Fields])) OR "therapy low level light"[All Fields] OR "Photobiomodulation Therapy"[All Fields] OR "Photobiomodulation Therapies"[All Fields] OR "therapies photobiomodulation"[All Fields] OR "therapy photobiomodulation"[All Fields] OR "LLLT"[All Fields] OR "laser therapy low level"[All Fields] OR ("low level light therapy"[MeSH Terms] OR ("low level"[All Fields] AND "light"[All Fields] AND "therapy"[All Fields]) OR "low level light therapy"[All Fields] OR ("laser"[All Fields] AND "therapies"[All Fields] AND "low"[All Fields] AND "level"[All Fields])) OR "laser therapy low level"[All Fields] OR "Low-Level Laser Therapies"[All Fields] OR ("low level light therapy"[MeSH Terms] OR ("low level"[All Fields] AND "light"[All Fields] AND "therapy"[All Fields]) OR "low level light therapy"[All Fields] OR ("laser"[All Fields] AND "irradiation"[All Fields] AND "low"[All Fields] AND "power"[All Fields])) OR ("low level light therapy"[MeSH Terms] OR ("low level"[All Fields] AND "light"[All Fields] AND "therapy"[All Fields]) OR "low level light therapy"[All Fields] OR ("irradiation"[All Fields] AND "low"[All Fields] AND "power"[All Fields] AND "laser"[All Fields])) OR ("low level light therapy"[MeSH Terms] OR ("low level"[All Fields] AND "light"[All Fields] AND "therapy"[All Fields]) OR "low level light therapy"[All Fields] OR ("laser"[All Fields] AND "irradiation"[All Fields] AND "low"[All Fields] AND "power"[All Fields])) OR "low power laser therapy"[All Fields] OR "low power laser therapy"[All Fields] OR "laser therapy low power"[All Fields] OR ("low level light therapy"[MeSH Terms] OR ("low level"[All Fields] AND "light"[All Fields] AND "therapy"[All Fields]) OR "low level light therapy"[All Fields] OR ("laser"[All Fields] AND "therapies"[All Fields] AND "low"[All Fields] AND "power"[All Fields])) OR "laser therapy low power"[All Fields] OR "Low-Power Laser Therapies"[All Fields] OR "low level laser therapy"[All Fields] OR "low level laser therapy"[All Fields] OR "low power laser irradiation"[All Fields] OR "low power laser irradiation"[All Fields] OR "Laser Biostimulation"[All Fields] OR "biostimulation laser"[All Fields] OR "Laser Phototherapy"[All Fields] OR "phototherapy laser"[All Fields] OR "Acupuncture"[All Fields] OR "Acupuncture"[MeSH Terms] OR "Pharmacopuncture"[All Fields] OR "Acupuncture Therapy"[All Fields] OR "Acupuncture Therapy"[MeSH Terms] OR "Acupuncture Treatment"[All Fields] OR "Acupuncture Treatments"[All Fields] OR "treatment acupuncture"[All Fields] OR "therapy acupuncture"[All Fields] OR "Pharmacoacupuncture Treatment"[All Fields] OR ("Acupuncture Therapy"[MeSH Terms] OR ("Acupuncture"[All Fields] AND "therapy"[All Fields]) OR "Acupuncture Therapy"[All Fields] OR ("treatment"[All Fields] AND "pharmacoacupuncture"[All Fields])) OR "Pharmacoacupuncture Therapy"[All Fields] OR ("Acupuncture Therapy"[MeSH Terms] OR ("Acupuncture"[All Fields] AND "therapy"[All Fields]) OR "Acupuncture Therapy"[All Fields] OR ("therapy"[All Fields] AND	

	"pharmacoacupuncture"[All Fields])) OR "Acupotomy"[All Fields] OR "Acupotomies"[All Fields]) AND ("Increased Salivary Flow"[All Fields] OR "Increased salivary volume"[All Fields] OR "Salivary flow"[All Fields] OR "saliva amount"[All Fields] OR "saliva quantity"[All Fields] OR "sialometry"[All Fields] OR "salivary flow rate"[All Fields] OR "salivary glands"[All Fields] OR "sialogogue"[All Fields] OR ("salivary"[All Fields] AND ("stimulate"[All Fields] OR "stimulated"[All Fields] OR "stimulates"[All Fields] OR "stimulating"[All Fields] OR "stimulation"[All Fields] OR "stimulations"[All Fields] OR "stimulative"[All Fields] OR "stimulator"[All Fields] OR "stimulator s"[All Fields] OR "stimulators"[All Fields])) OR "salivations"[All Fields] OR "parotid salivary flow"[All Fields] OR "unstimulated saliva flow"[All Fields] OR "salivary function"[All Fields] OR "secretory rate"[All Fields] OR "sublingual gland"[All Fields] OR "submandibular gland"[All Fields])	
Livivo	("head and neck neoplasms" OR "Neoplasms, Head and Neck" OR "head-and-neck cancer" OR "Head, Neck Neoplasms" OR "Head and Neck Neoplasm" OR "Cancer of Head and Neck" OR "Head and Neck Cancer" OR "Cancer of the Head and Neck" OR "Head Neoplasms" OR "Neoplasms, Head" OR "Head Neoplasm" OR "Neoplasm, Head" OR "Neck Neoplasms" OR "Neoplasms, Neck" OR "Neck Neoplasm" OR "Neoplasm, Neck" OR "Cancer of Head" OR "Head Cancers" OR "Head Cancer" OR "Cancer, Head" OR "Cancers, Head" OR "Cancer of the Head" OR "Cancer of Neck" OR "Neck Cancers" OR "Neck Cancer" OR "Cancer, Neck" OR "Cancers, Neck" OR "Cancer of the Neck" OR "radiotherapy" OR "Radiotherapies" OR "Radiation Therapy" OR "intensity-modulated radiotherapy" OR "Radiation Therapies" OR "Therapies, Radiation" OR "Therapy, Radiation" OR "Radiation Treatment" OR "Radiation Treatments" OR "Treatment, Radiation" OR "Radiotherapy, Targeted" OR "Radiotherapies, Targeted" OR "Targeted Radiotherapies" OR "Targeted Radiotherapy" OR "Targeted Radiation Therapy" OR "Radiation Therapies, Targeted" OR "Radiation Dosage" OR "Iodine Radioisotopes" OR "Targeted Radiation Therapies" OR "Therapies, Targeted Radiation" OR "Therapy, Targeted Radiation" OR "Radiation Therapy, Targeted" OR "Radiation Injuries" OR "radiation effects" OR "intensity-modulated radiotherapy" OR "cancer complications" OR "Sjogren's Syndrome" OR "Sjogrens Syndrome" OR "Syndrome, Sjogren's" OR "Sjogren Syndrome" OR "Sicca Syndrome" OR "Syndrome, Sicca" OR "Diabetes Mellitus" OR "Diabetes Insipidus" OR "Diet, Diabetic" OR "Prediabetic State" OR "Scleredema Adultorum" OR "Glycation End Products, Advanced" OR "Glucose Intolerance" OR "Gastroparesis" OR "Antihypertensive Agents" OR "Agents, Antihypertensive" OR "Antihypertensive Agent" OR "Agent, Antihypertensive" OR "Anti-Hypertensive Agent" OR "Agent, Anti-Hypertensive" OR "Anti Hypertensive Agent" OR "Anti-Hypertensive Drug" OR "Anti Hypertensive Drug" OR "Drug, Anti-Hypertensive" OR "Antihypertensive Drug" OR "Drug, Antihypertensive" OR "Antihypertensives" OR "Anti-Hypertensive Agents" OR "Agents, Anti-Hypertensive" OR "Anti Hypertensive Agents" OR "Anti-Hypertensive Drugs" OR "Anti Hypertensive Drugs" OR "Drugs, Anti-Hypertensive" OR "Anti-Hypertensives" OR "Anti Hypertensives" OR "Antihypertensive Drugs" OR "Drugs, Antihypertensive" OR "Anti-Hypertensive" OR "Anti Hypertensive" OR "Antihypertensive" OR "Cystic Fibrosis" OR "Fibrosis, Cystic" OR "Mucoviscidosis" OR "Pulmonary Cystic Fibrosis" OR "Cystic Fibrosis, Pulmonary" OR "Pancreatic Cystic Fibrosis" OR "Cystic Fibrosis, Pancreatic" OR "Fibrocystic Disease of Pancreas" OR "Pancreas Fibrocystic Disease" OR "Pancreas Fibrocystic Diseases" OR "Cystic Fibrosis of Pancreas" OR "Cholinergic Antagonists" OR "Antagonists, Cholinergic" OR "Cholinergic Receptor Antagonist" OR "Antagonist, Cholinergic Receptor" OR "Receptor Antagonist, Cholinergic" OR "Cholinergic-Blocking Agent" OR "Agent, Cholinergic-Blocking" OR "Cholinergic Blocking Agent" OR "Cholinolytic" OR "Cholinolytics" OR "Acetylcholine Antagonist" OR "Antagonist, Acetylcholine" OR "Cholinergic Antagonist" OR "Antagonist, Cholinergic" OR "Cholinergic Receptor Antagonists" OR "Antagonists, Cholinergic Receptor" OR "Receptor Antagonists, Cholinergic" OR "Cholinergic-Blocking Agents" OR "Agents, Cholinergic-Blocking" OR "Cholinergic Blocking Agents" OR "Acetylcholine Antagonists" OR "Antagonists, Acetylcholine" OR "Anticholinergic Agents" OR "Agents, Anticholinergic" OR "Anticholinergic Agent" OR "Agent, Anticholinergic" OR "Anticholinergics" OR "Anti-Cholinergics" OR "Anti Cholinergics" OR "Anti-Cholinergic" OR "Anti Cholinergic" OR "Anticholinergic" OR "Histamine Antagonists" OR "Antagonists, Histamine" OR "Antihistamines" OR "Histamine Antagonist" OR "Antagonist, Histamine" OR "Antihistamine" OR "Antidepressive Agents" OR "Agents, Antidepressive" OR "Antidepressive Agent" OR "Agent, Antidepressive" OR "Antidepressants" OR "Antidepressant" OR "Antidepressant Drugs" OR "Drugs, Antidepressant" OR "Antidepressant Drug" OR "Drug, Antidepressant" OR "Thymoleptics" OR "Thymoleptic" OR "Thymoanaleptics" OR "Thymoanaleptic" OR "Antiparkinson Agents" OR "Agents, Antiparkinson" OR "Antiparkinsonian Agents" OR "Agents, Antiparkinsonian" OR "Antiparkinsonians" OR "Antiparkinson Drugs" OR "Drugs, Antiparkinson" OR "Anti-Anxiety Agents" OR "Agents, Anti-Anxiety" OR "Anti Anxiety Agents" OR "Anti-Anxiety Drug" OR "Anti Anxiety Drug" OR "Drug, Anti-Anxiety" OR "Anti-Anxiety Drugs" OR "Anti Anxiety Drugs" OR "Drugs, Anti-Anxiety" OR "Anxiolytic" OR "Anxiolytics" OR "Tranquilizing Agents, Minor" OR "Agents, Minor Tranquilizing" OR "Minor Tranquilizing Agents" OR "Tranquillizing Agents, Minor" OR "Agents, Minor Tranquillizing" OR "Minor Tranquillizing Agents" OR "Anxiolytic Agent" OR "Agent, Anxiolytic" OR "Anti-Anxiety Agent" OR "Agent, Anti-Anxiety" OR "Anti Anxiety Agent" OR "Anxiolytic Agents" OR "Agents, Anxiolytic" OR "Anti-Anxiety Effect" OR "Anti Anxiety Effect" OR "Effect, Anti-Anxiety" OR "Anxiolytic Effects" OR "Effects, Anxiolytic" OR "Antianxiety Effects" OR "Effects, Antianxiety" OR "Anxiolytic Effect" OR "Effect, Anxiolytic" OR "Anti-Anxiety Effects" OR "Anti Anxiety Effects" OR "Effects, Anti-Anxiety" OR "Antianxiety Effect" OR "Effect, Antianxiety" OR "Obesity" OR "xerostomia" OR "Xerostomias" OR "xerostomy" OR "Hyposalivation" OR "Hyposalivations" OR "Asialia" OR "Salivary gland dysfunction" OR "Mouth Dryness" OR "Dryness, Mouth" OR "oral dryness" OR "Salivary hypo-function" OR "dry mouth" OR "Radiation-induced xerostomia") AND ("Transcutaneous Electric Nerve Stimulation" OR "Electric Stimulation, Transcutaneous" OR "Stimulation, Transcutaneous Electric" OR "Transcutaneous Electric Stimulation" OR "Percutaneous Electric Nerve Stimulation" OR "TENS" OR "Electrical Stimulation, Transcutaneous" OR "Transcutaneous Electrical Stimulation" OR "Transdermal Electrostimulation" OR	64

	"Electrostimulation, Transdermal" OR "Percutaneous Electrical Nerve Stimulation" OR "Transcutaneous Electrical Nerve Stimulation" OR "Transcutaneous Nerve Stimulation" OR "Nerve Stimulation, Transcutaneous" OR "Stimulation, Transcutaneous Nerve" OR "Percutaneous Neuromodulation Therapy" OR "Neuromodulation Therapy, Percutaneous" OR "Percutaneous Neuromodulation Therapies" OR "Therapy, Percutaneous Neuromodulation" OR "Percutaneous Electrical Neuromodulation" OR "Electrical Neuromodulation, Percutaneous" OR "Electrical Neuromodulations, Percutaneous" OR "Neuromodulation, Percutaneous Electrical" OR "Neuromodulations, Percutaneous Electrical" OR "Percutaneous Electrical Neuromodulations" OR "Chewing Gums" OR "Chewing Gum" OR "Gum, Chewing" OR "Gums, Chewing" OR "Chewing" OR "Low-Level Light Therapy" OR "Light Therapies, Low-Level" OR "Light Therapy, Low-Level" OR "Low Level Light Therapy" OR "Low-Level Light Therapies" OR "Therapies, Low-Level Light" OR "Therapy, Low-Level Light" OR "Photobiomodulation Therapy" OR "Photobiomodulation Therapies" OR "Therapies, Photobiomodulation" OR "Therapy, Photobiomodulation" OR "LLLT" OR "Laser Therapy, Low-Level" OR "Laser Therapies, Low-Level" OR "Laser Therapy, Low Level" OR "Low-Level Laser Therapies" OR "Laser Irradiation, Low-Power" OR "Irradiation, Low-Power Laser" OR "Laser Irradiation, Low Power" OR "Low-Power Laser Therapy" OR "Low Power Laser Therapy" OR "Laser Therapy, Low-Power" OR "Laser Therapies, Low-Power" OR "Laser Therapy, Low Power" OR "Low-Power Laser Therapies" OR "Low-Level Laser Therapy" OR "Low Level Laser Therapy" OR "Low-Power Laser Irradiation" OR "Low Power Laser Irradiation" OR "Laser Biostimulation" OR "Biostimulation, Laser" OR "Laser Phototherapy" OR "Phototherapy, Laser" OR "Acupuncture" OR "Pharmacopuncture" OR "acupuncture therapy" OR "Acupuncture Treatment" OR "Acupuncture Treatments" OR "Treatment, Acupuncture" OR "Therapy, Acupuncture" OR "Pharmacoacupuncture Treatment" OR "Treatment, Pharmacoacupuncture" OR "Pharmacoacupuncture Therapy" OR "Therapy, Pharmacoacupuncture" OR "Acupotomy" OR "Acupotomies") AND ("Increased Salivary Flow" OR "Increased salivary volume" OR "Salivary flow" OR "saliva amount" OR "saliva quantity" OR "sialometry" OR "salivary flow rate" OR "salivary glands" OR "sialogogue" OR "salivary stimulations" OR "salivations" OR "parotid salivary flow" OR "unstimulated saliva flow" OR "salivary function" OR "secretory rate" OR "sublingual gland" OR "submandibular gland")	
Scopus	TITLE-ABS-KEY (("head and neck neoplasms" OR "Neoplasms, Head and Neck" OR "head-and-neck cancer" OR "Head, Neck Neoplasms" OR "Head and Neck Neoplasm" OR "Cancer of Head and Neck" OR "Head and Neck Cancer" OR "Cancer of the Head and Neck" OR "Head Neoplasms" OR "Neoplasms, Head" OR "Head Neoplasm" OR "Neoplasm, Head" OR "Neck Neoplasms" OR "Neoplasms, Neck" OR "Neck Neoplasm" OR "Neoplasm, Neck" OR "Cancer of Head" OR "Head Cancers" OR "Head Cancer" OR "Cancer, Head" OR "Cancers, Head" OR "Cancer of the Head" OR "Cancer of Neck" OR "Neck Cancers" OR "Neck Cancer" OR "Cancer, Neck" OR "Cancers, Neck" OR "Cancer of the Neck" OR "radiotherapy" OR "Radiotherapies" OR "Radiation Therapy" OR "intensity-modulated radiotherapy" OR "Radiation Therapies" OR "Therapies, Radiation" OR "Therapy, Radiation" OR "Radiation Treatment" OR "Radiation Treatments" OR "Treatment, Radiation" OR "Radiotherapy, Targeted" OR "Radiotherapies, Targeted" OR "Targeted Radiotherapies" OR "Targeted Radiotherapy" OR "Targeted Radiation Therapy" OR "Radiation Therapies, Targeted" OR "Radiation Dosage" OR "Iodine Radioisotopes" OR "Targeted Radiation Therapies" OR "Therapies, Targeted Radiation" OR "Therapy, Targeted Radiation" OR "Radiation Therapy, Targeted" OR "Radiation Injuries" OR "radiation effects" OR "intensity-modulated radiotherapy" OR "cancer complications" OR "Sjogren's Syndrome" OR "Sjogrens Syndrome" OR "Syndrome, Sjogren's" OR "Sjogren Syndrome" OR "Sicca Syndrome" OR "Syndrome, Sicca" OR "Diabetes Mellitus" OR "Diabetes Insipidus" OR "Diet, Diabetic" OR "Prediabetic State" OR "Scleredema Adultorum" OR "Glycation End Products, Advanced" OR "Glucose Intolerance" OR "Gastroparesis" OR "Antihypertensive Agents" OR "Agents, Antihypertensive" OR "Antihypertensive Agent" OR "Agent, Antihypertensive" OR "Anti-Hypertensive Agent" OR "Agent, Anti-Hypertensive" OR "Anti Hypertensive Agent" OR "Anti-Hypertensive Drug" OR "Anti Hypertensive Drug" OR "Drug, Anti-Hypertensive" OR "Antihypertensive Drug" OR "Drug, Antihypertensive" OR "Antihypertensives" OR "Anti-Hypertensive Agents" OR "Agents, Anti-Hypertensive" OR "Anti Hypertensive Agents" OR "Anti-Hypertensive Drugs" OR "Anti Hypertensive Drugs" OR "Drugs, Anti-Hypertensive" OR "Anti-Hypertensives" OR "Anti Hypertensives" OR "Antihypertensive Drugs" OR "Drugs, Antihypertensive" OR "Anti-Hypertensive" OR "Anti Hypertensive" OR "Antihypertensive" OR "Cystic Fibrosis" OR "Fibrosis, Cystic" OR "Mucoviscidosis" OR "Pulmonary Cystic Fibrosis" OR "Cystic Fibrosis, Pulmonary" OR "Pancreatic Cystic Fibrosis" OR "Cystic Fibrosis, Pancreatic" OR "Fibrocystic Disease of Pancreas" OR "Pancreas Fibrocystic Disease" OR "Pancreas Fibrocystic Diseases" OR "Cystic Fibrosis of Pancreas" OR "Cholinergic Antagonists" OR "Antagonists, Cholinergic" OR "Cholinergic Receptor Antagonist" OR "Antagonist, Cholinergic Receptor" OR "Receptor Antagonist, Cholinergic" OR "Cholinergic-Blocking Agent" OR "Agent, Cholinergic-Blocking" OR "Cholinergic Blocking Agent" OR "Cholinolytic" OR "Cholinolytics" OR "Acetylcholine Antagonist" OR "Antagonist, Acetylcholine" OR "Cholinergic Antagonist" OR "Antagonist, Cholinergic" OR "Cholinergic Receptor Antagonists" OR "Antagonists, Cholinergic Receptor" OR "Receptor Antagonists, Cholinergic" OR "Cholinergic-Blocking Agents" OR "Agents, Cholinergic-Blocking" OR "Cholinergic Blocking Agents" OR "Acetylcholine Antagonists" OR "Antagonists, Acetylcholine" OR "Anticholinergic Agents" OR "Agents, Anticholinergic" OR "Anticholinergic Agent" OR "Agent, Anticholinergic" OR "Anticholinergics" OR "Anti-Cholinergics" OR "Anti Cholinergics" OR "Anti-Cholinergic" OR "Anti Cholinergic" OR "Anticholinergic" OR "Histamine Antagonists" OR "Antagonists, Histamine" OR "Antihistamines" OR "Histamine Antagonist" OR "Antagonist,	489

	Histamine" OR "Antihistamine" OR "Antidepressive Agents" OR "Agents, Antidepressive" OR "Antidepressive Agent" OR "Agent, Antidepressive" OR "Antidepressants" OR "Antidepressant" OR "Antidepressant Drugs" OR "Drugs, Antidepressant" OR "Antidepressant Drug" OR "Drug, Antidepressant" OR "Thymoleptics" OR "Thymoleptic" OR "Thymoanaleptics" OR "Thymoanaleptic" OR "Antiparkinson Agents" OR "Agents, Antiparkinson" OR "Antiparkinsonian Agents" OR "Agents, Antiparkinsonian" OR "Antiparkinsonians" OR "Antiparkinson Drugs" OR "Drugs, Antiparkinson" OR "Anti-Anxiety Agents" OR "Agents, Anti-Anxiety" OR "Anti Anxiety Agents" OR "Anti-Anxiety Drug" OR "Anti Anxiety Drug" OR "Drug, Anti-Anxiety" OR "Anti-Anxiety Drugs" OR "Anti Anxiety Drugs" OR "Drugs, Anti-Anxiety" OR "Anxiolytic" OR "Anxiolytics" OR "Tranquilizing Agents, Minor" OR "Agents, Minor Tranquilizing" OR "Minor Tranquilizing Agents" OR "Tranquillizing Agents, Minor" OR "Agents, Minor Tranquillizing" OR "Minor Tranquillizing Agents" OR "Anxiolytic Agent" OR "Agent, Anxiolytic" OR "Anti-Anxiety Agent" OR "Agent, Anti-Anxiety" OR "Anti Anxiety Agent" OR "Anxiolytic Agents" OR "Agents, Anxiolytic" OR "Anti-Anxiety Effect" OR "Anti Anxiety Effect" OR "Effect, Anti-Anxiety" OR "Anxiolytic Effects" OR "Effects, Anxiolytic" OR "Antianxiety Effects" OR "Effects, Antianxiety" OR "Anxiolytic Effect" OR "Effect, Anxiolytic" OR "Anti-Anxiety Effects" OR "Anti Anxiety Effects" OR "Effects, Anti-Anxiety" OR "Antianxiety Effect" OR "Effect, Antianxiety" OR "Obesity" OR "xerostomia" OR "Xerostomias" OR "xerostomy" OR "Hyposalivation" OR "Hyposalivations" OR "Asialia" OR "Salivary gland dysfunction" OR "Mouth Dryness" OR "Dryness, Mouth" OR "oral dryness" OR "Salivary hypo-function" OR "dry mouth" OR "Radiation-induced xerostomia") AND ("Transcutaneous Electric Nerve Stimulation" OR "Electric Stimulation, Transcutaneous" OR "Stimulation, Transcutaneous Electric" OR "Transcutaneous Electric Stimulation" OR "Percutaneous Electric Nerve Stimulation" OR "TENS" OR "Electrical Stimulation, Transcutaneous" OR "Transcutaneous Electrical Stimulation" OR "Transdermal Electrostimulation" OR "Electrostimulation, Transdermal" OR "Percutaneous Electrical Nerve Stimulation" OR "Transcutaneous Electrical Nerve Stimulation" OR "Transcutaneous Nerve Stimulation" OR "Nerve Stimulation, Transcutaneous" OR "Stimulation, Transcutaneous Nerve" OR "Percutaneous Neuromodulation Therapy" OR "Neuromodulation Therapy, Percutaneous" OR "Percutaneous Neuromodulation Therapies" OR "Therapy, Percutaneous Neuromodulation" OR "Percutaneous Electrical Neuromodulation" OR "Electrical Neuromodulation, Percutaneous" OR "Electrical Neuromodulations, Percutaneous" OR "Neuromodulation, Percutaneous Electrical" OR "Neuromodulations, Percutaneous Electrical" OR "Percutaneous Electrical Neuromodulations" OR "Chewing Gums" OR "Chewing Gum" OR "Gum, Chewing" OR "Gums, Chewing" OR "Chewing" OR "Low-Level Light Therapy" OR "Light Therapies, Low-Level" OR "Light Therapy, Low-Level" OR "Low Level Light Therapy" OR "Low-Level Light Therapies" OR "Therapies, Low-Level Light" OR "Therapy, Low-Level Light" OR "Photobiomodulation Therapy" OR "Photobiomodulation Therapies" OR "Therapies, Photobiomodulation" OR "Therapy, Photobiomodulation" OR "LLLT" OR "Laser Therapy, Low-Level" OR "Laser Therapies, Low-Level" OR "Laser Therapy, Low Level" OR "Low-Level Laser Therapies" OR "Laser Irradiation, Low-Power" OR "Irradiation, Low-Power Laser" OR "Laser Irradiation, Low Power" OR "Low-Power Laser Therapy" OR "Low Power Laser Therapy" OR "Laser Therapy, Low-Power" OR "Laser Therapies, Low-Power" OR "Laser Therapy, Low Power" OR "Low-Power Laser Therapies" OR "Low-Level Laser Therapy" OR "Low Level Laser Therapy" OR "Low-Power Laser Irradiation" OR "Low Power Laser Irradiation" OR "Laser Biostimulation" OR "Biostimulation, Laser" OR "Laser Phototherapy" OR "Phototherapy, Laser" OR "Acupuncture" OR "Pharmacopuncture" OR "acupuncture therapy" OR "Acupuncture Treatment" OR "Acupuncture Treatments" OR "Treatment, Acupuncture" OR "Therapy, Acupuncture" OR "Pharmacoacupuncture Treatment" OR "Treatment, Pharmacoacupuncture" OR "Pharmacoacupuncture Therapy" OR "Therapy, Pharmacoacupuncture" OR "Acupotomy" OR "Acupotomies") AND ("Increased Salivary Flow" OR "Increased salivary volume" OR "Salivary flow" OR "saliva amount" OR "saliva quantity" OR "sialometry" OR "salivary flow rate" OR "salivary glands" OR "sialogogue" OR "salivary stimulations" OR "salivations" OR "parotid salivary flow" OR "unstimulated saliva flow" OR "salivary function" OR "secretory rate" OR "sublingual gland" OR "submandibular gland"))	
Embase	('head and neck neoplasms'/exp OR 'head and neck neoplasms' OR 'neoplasms, head and neck' OR 'head-and-neck cancer'/exp OR 'head-and-neck cancer' OR 'head, neck neoplasms' OR 'head and neck neoplasm' OR 'cancer of head and neck' OR 'head and neck cancer'/exp OR 'head and neck cancer' OR 'cancer of the head and neck' OR 'head neoplasms'/exp OR 'head neoplasms' OR 'neoplasms, head' OR 'head neoplasm'/exp OR 'head neoplasm' OR 'neoplasm, head' OR 'neck neoplasms'/exp OR 'neck neoplasms' OR 'neoplasms, neck' OR 'neck neoplasm' OR 'neoplasm, neck' OR 'cancer of head' OR 'head cancers' OR 'head cancer'/exp OR 'head cancer' OR 'cancer, head'/exp OR 'cancer, head' OR 'cancers, head' OR 'cancer of the head' OR 'cancer of neck' OR 'neck cancers' OR 'neck cancer'/exp OR 'neck cancer' OR 'cancer, neck' OR 'cancers, neck' OR 'cancer of the neck' OR 'radiotherapy'/exp OR 'radiotherapy' OR 'radiotherapies' OR 'radiation therapy'/exp OR 'radiation therapy' OR 'radiation therapies' OR 'therapies, radiation' OR 'therapy, radiation'/exp OR 'therapy, radiation' OR 'radiation treatment'/exp OR 'radiation treatment' OR 'radiation treatments' OR 'treatment, radiation'/exp OR 'treatment, radiation' OR 'radiotherapy, targeted' OR 'radiotherapies, targeted' OR 'targeted radiotherapies' OR 'targeted radiotherapy' OR 'targeted radiation therapy' OR 'radiation therapies, targeted' OR 'radiation dosage'/exp OR 'radiation dosage' OR 'iodine radioisotopes'/exp OR 'iodine radioisotopes' OR 'targeted radiation therapies' OR 'therapies, targeted radiation' OR 'therapy, targeted radiation' OR 'radiation therapy, targeted' OR 'radiation injuries'/exp OR 'radiation injuries' OR 'radiation effects'/exp OR 'radiation effects' OR 'intensity-modulated radiotherapy'/exp OR 'intensity-modulated radiotherapy' OR 'cancer complications' OR 'sjogrens syndrome' OR 'syndrome, sjogrens' OR 'sjogren syndrome'/exp OR 'sjogren syndrome' OR 'sicca syndrome'/exp OR 'sicca syndrome' OR 'syndrome, sicca' OR 'diabetes mellitus'/exp OR 'diabetes mellitus' OR 'diabetes insipidus'/exp OR	677

	'diabetes insipidus' OR 'diet, diabetic'/exp OR 'diet, diabetic' OR 'prediabetic state'/exp OR 'prediabetic state' OR 'scleredema adultorum'/exp OR 'scleredema adultorum' OR 'glycation end products, advanced'/exp OR 'glycation end products, advanced' OR 'glucose intolerance'/exp OR 'glucose intolerance' OR 'gastroparesis'/exp OR 'gastroparesis' OR 'antihypertensive agents'/exp OR 'antihypertensive agents' OR 'agents, antihypertensive' OR 'antihypertensive agent'/exp OR 'antihypertensive agent' OR 'agent, antihypertensive' OR 'anti-hypertensive agent'/exp OR 'anti-hypertensive agent' OR 'agent, anti-hypertensive' OR 'anti hypertensive agent'/exp OR 'anti hypertensive agent' OR 'anti-hypertensive drug'/exp OR 'anti-hypertensive drug' OR 'anti hypertensive drug'/exp OR 'anti hypertensive drug' OR 'drug, anti-hypertensive' OR 'antihypertensive drug'/exp OR 'antihypertensive drug' OR 'drug, antihypertensive' OR 'antihypertensives'/exp OR 'antihypertensives' OR 'anti-hypertensive agents' OR 'agents, anti-hypertensive' OR 'anti hypertensive agents' OR 'anti-hypertensive drugs' OR 'anti hypertensive drugs' OR 'drugs, anti-hypertensive' OR 'anti-hypertensives' OR 'anti hypertensives' OR 'antihypertensive drugs' OR 'drugs, antihypertensive' OR 'anti-hypertensive'/exp OR 'anti-hypertensive' OR 'anti hypertensive'/exp OR 'anti hypertensive' OR 'antihypertensive'/exp OR 'antihypertensive' OR 'cystic fibrosis'/exp OR 'cystic fibrosis' OR 'fibrosis, cystic' OR 'mucoviscidosis'/exp OR 'mucoviscidosis' OR 'pulmonary cystic fibrosis' OR 'cystic fibrosis, pulmonary' OR 'pancreatic cystic fibrosis'/exp OR 'pancreatic cystic fibrosis' OR 'cystic fibrosis, pancreatic' OR 'fibrocystic disease of pancreas' OR 'pancreas fibrocystic disease'/exp OR 'pancreas fibrocystic disease' OR 'pancreas fibrocystic diseases' OR 'cystic fibrosis of pancreas'/exp OR 'cystic fibrosis of pancreas' OR 'cholinergic antagonists'/exp OR 'cholinergic antagonists' OR 'antagonists, cholinergic' OR 'cholinergic receptor antagonist'/exp OR 'cholinergic receptor antagonist' OR 'antagonist, cholinergic receptor' OR 'receptor antagonist, cholinergic' OR 'cholinergic-blocking agent'/exp OR 'cholinergic-blocking agent' OR 'agent, cholinergic-blocking' OR 'cholinergic blocking agent'/exp OR 'cholinergic blocking agent' OR 'cholinolytic' OR 'cholinolytics' OR 'acetylcholine antagonist'/exp OR 'acetylcholine antagonist' OR 'antagonist, acetylcholine' OR 'cholinergic antagonist'/exp OR 'cholinergic antagonist' OR 'antagonist, cholinergic' OR 'cholinergic receptor antagonists' OR 'antagonists, cholinergic receptor' OR 'receptor antagonists, cholinergic' OR 'cholinergic-blocking agents' OR 'agents, cholinergic-blocking' OR 'cholinergic blocking agents' OR 'acetylcholine antagonists'/exp OR 'acetylcholine antagonists' OR 'antagonists, acetylcholine' OR 'anticholinergic agents' OR 'agents, anticholinergic' OR 'anticholinergic agent'/exp OR 'anticholinergic agent' OR 'agent, anticholinergic' OR 'anticholinergics'/exp OR 'anticholinergics' OR 'anti-cholinergics' OR 'anti cholinergics' OR 'anti-cholinergic' OR 'anti cholinergic' OR 'anticholinergic'/exp OR 'anticholinergic' OR 'histamine antagonists'/exp OR 'histamine antagonists' OR 'antagonists, histamine' OR 'antihistamines'/exp OR 'antihistamines' OR 'histamine antagonist'/exp OR 'histamine antagonist' OR 'antagonist, histamine' OR 'antihistamine'/exp OR 'antihistamine' OR 'antidepressive agents'/exp OR 'antidepressive agents' OR 'agents, antidepressive' OR 'antidepressive agent'/exp OR 'antidepressive agent' OR 'agent, antidepressive' OR 'antidepressants'/exp OR 'antidepressants' OR 'antidepressant'/exp OR 'antidepressant' OR 'antidepressant drugs' OR 'drugs, antidepressant' OR 'antidepressant drug'/exp OR 'antidepressant drug' OR 'drug, antidepressant' OR 'thymoleptics' OR 'thymoleptic'/exp OR 'thymoleptic' OR 'thymoanaleptics' OR 'thymoanaleptic' OR 'antiparkinson agents'/exp OR 'antiparkinson agents' OR 'agents, antiparkinson' OR 'antiparkinsonian agents' OR 'agents, antiparkinsonian' OR 'antiparkinsonians' OR 'antiparkinson drugs' OR 'drugs, antiparkinson' OR 'anti-anxiety agents'/exp OR 'anti-anxiety agents' OR 'agents, anti-anxiety' OR 'anti anxiety agents'/exp OR 'anti anxiety agents' OR 'anti-anxiety drug' OR 'anti anxiety drug' OR 'drug, anti-anxiety' OR 'anti-anxiety drugs' OR 'anti anxiety drugs' OR 'drugs, anti-anxiety' OR 'anxiolytic'/exp OR 'anxiolytic' OR 'anxiolytics'/exp OR 'anxiolytics' OR 'tranquilizing agents, minor'/exp OR 'tranquilizing agents, minor' OR 'agents, minor tranquilizing' OR 'minor tranquilizing agents' OR 'tranquillizing agents, minor' OR 'agents, minor tranquillizing' OR 'minor tranquillizing agents' OR 'anxiolytic agent'/exp OR 'anxiolytic agent' OR 'agent, anxiolytic' OR 'anti-anxiety agent' OR 'agent, anti-anxiety' OR 'anti anxiety agent' OR 'anxiolytic agents' OR 'agents, anxiolytic' OR 'anti-anxiety effect' OR 'anti anxiety effect' OR 'effect, anti-anxiety' OR 'anxiolytic effects' OR 'effects, anxiolytic' OR 'antianxiety effects' OR 'effects, antianxiety' OR 'anxiolytic effect' OR 'effect, anxiolytic' OR 'anti-anxiety effects' OR 'anti anxiety effects' OR 'effects, anti-anxiety' OR 'antianxiety effect' OR 'effect, antianxiety' OR 'obesity'/exp OR 'obesity' OR 'xerostomia'/exp OR 'xerostomia' OR 'xerostomias' OR 'xerostomy'/exp OR 'xerostomy' OR 'hyposalivation'/exp OR 'hyposalivation' OR 'hyposalivations' OR 'asialia' OR 'salivary gland dysfunction'/exp OR 'salivary gland dysfunction' OR 'mouth dryness'/exp OR 'mouth dryness' OR 'dryness, mouth' OR 'oral dryness'/exp OR 'oral dryness' OR 'salivary hypo-function' OR 'dry mouth'/exp OR 'dry mouth' OR 'radiation-induced xerostomia') AND ('transcutaneous electric nerve stimulation'/exp OR 'transcutaneous electric nerve stimulation' OR 'electric stimulation, transcutaneous' OR 'stimulation, transcutaneous electric' OR 'transcutaneous electric stimulation' OR 'percutaneous electric nerve stimulation'/exp OR 'percutaneous electric nerve stimulation' OR 'tens' OR 'electrical stimulation, transcutaneous' OR 'transcutaneous electrical stimulation'/exp OR 'transcutaneous electrical stimulation' OR 'transdermal electrostimulation' OR 'electrostimulation, transdermal' OR 'percutaneous electrical nerve stimulation'/exp OR 'percutaneous electrical nerve stimulation' OR 'transcutaneous electrical nerve stimulation'/exp OR 'transcutaneous electrical nerve stimulation' OR 'transcutaneous nerve stimulation'/exp OR 'transcutaneous nerve stimulation' OR 'nerve stimulation, transcutaneous'/exp OR 'nerve stimulation, transcutaneous' OR 'stimulation, transcutaneous nerve' OR 'percutaneous neuromodulation therapy' OR 'neuromodulation therapy, percutaneous' OR 'percutaneous neuromodulation therapies' OR 'therapy, percutaneous neuromodulation' OR 'percutaneous electrical neuromodulation' OR 'electrical neuromodulation, percutaneous' OR 'electrical neuromodulations, percutaneous' OR 'neuromodulation, percutaneous electrical' OR 'neuromodulations, percutaneous electrical' OR 'percutaneous electrical neuromodulations' OR 'chewing gums' OR 'chewing gum'/exp OR 'chewing gum' OR 'gum, chewing' OR 'gums, chewing' OR 'chewing'/exp OR 'chewing' OR 'low-level light therapy'/exp OR 'low-level light therapy' OR 'light	

	therapies, low-level' OR 'light therapy, low-level' OR 'low level light therapy'/exp OR 'low level light therapy' OR 'low-level light therapies' OR 'therapies, low-level light' OR 'therapy, low-level light' OR 'photobiomodulation therapy'/exp OR 'photobiomodulation therapy' OR 'photobiomodulation therapies' OR 'therapies, photobiomodulation' OR 'therapy, photobiomodulation' OR 'lllt' OR 'laser therapy, low-level'/exp OR 'laser therapy, low-level' OR 'laser therapies, low-level' OR 'laser therapy, low level'/exp OR 'laser therapy, low level' OR 'low-level laser therapies' OR 'laser irradiation, low-power' OR 'irradiation, low-power laser' OR 'laser irradiation, low power' OR 'low-power laser therapy'/exp OR 'low-power laser therapy' OR 'low power laser therapy'/exp OR 'low power laser therapy' OR 'laser therapy, low-power' OR 'laser therapies, low-power' OR 'laser therapy, low power' OR 'low-power laser therapies' OR 'low-level laser therapy'/exp OR 'low-level laser therapy' OR 'low level laser therapy'/exp OR 'low level laser therapy' OR 'low-power laser irradiation' OR 'low power laser irradiation'/exp OR 'low power laser irradiation' OR 'laser biostimulation'/exp OR 'laser biostimulation' OR 'biostimulation, laser' OR 'laser phototherapy' OR 'phototherapy, laser' OR 'acupuncture'/exp OR 'acupuncture' OR 'pharmacopuncture'/exp OR 'pharmacopuncture' OR 'acupuncture therapy'/exp OR 'acupuncture therapy' OR 'acupuncture treatment' OR 'acupuncture treatments' OR 'treatment, acupuncture' OR 'therapy, acupuncture' OR 'pharmacoacupuncture treatment' OR 'treatment, pharmacoacupuncture' OR 'pharmacoacupuncture therapy' OR 'therapy, pharmacoacupuncture' OR 'acupotomy'/exp OR 'acupotomy' OR 'acupotomies') AND ('increased salivary flow' OR 'increased salivary volume' OR 'salivary flow'/exp OR 'salivary flow' OR 'saliva amount' OR 'saliva quantity' OR 'sialometry'/exp OR 'sialometry' OR 'salivary flow rate'/exp OR 'salivary flow rate' OR 'salivary glands'/exp OR 'salivary glands' OR 'sialogogue' OR 'salivary stimulations' OR 'salivations' OR 'parotid salivary flow' OR 'unstimulated saliva flow' OR 'salivary function' OR 'secretory rate'/exp OR 'secretory rate' OR 'sublingual gland'/exp OR 'sublingual gland' OR 'submandibular gland'/exp OR 'submandibular gland')	
Proquest	noft(("head and neck neoplasms" OR "Neoplasms, Head and Neck" OR "head-and-neck cancer" OR "Head, Neck Neoplasms" OR "Head and Neck Neoplasm" OR "Cancer of Head and Neck" OR "Head and Neck Cancer" OR "Cancer of the Head and Neck" OR "Head Neoplasms" OR "Neoplasms, Head" OR "Head Neoplasm" OR "Neoplasm, Head" OR "Neck Neoplasms" OR "Neoplasms, Neck" OR "Neck Neoplasm" OR "Neoplasm, Neck" OR "Cancer of Head" OR "Head Cancers" OR "Head Cancer" OR "Cancer, Head" OR "Cancers, Head" OR "Cancer of the Head" OR "Cancer of Neck" OR "Neck Cancers" OR "Neck Cancer" OR "Cancer, Neck" OR "Cancers, Neck" OR "Cancer of the Neck" OR "radiotherapy" OR "Radiotherapies" OR "Radiation Therapy" OR "intensity-modulated radiotherapy" OR "Radiation Therapies" OR "Therapies, Radiation" OR "Therapy, Radiation" OR "Radiation Treatment" OR "Radiation Treatments" OR "Treatment, Radiation" OR "Radiotherapy, Targeted" OR "Radiotherapies, Targeted" OR "Targeted Radiotherapies" OR "Targeted Radiotherapy" OR "Targeted Radiation Therapy" OR "Radiation Therapies, Targeted" OR "Radiation Dosage" OR "Iodine Radioisotopes" OR "Targeted Radiation Therapies" OR "Therapies, Targeted Radiation" OR "Therapy, Targeted Radiation" OR "Radiation Therapy, Targeted" OR "Radiation Injuries" OR "radiation effects" OR "intensity-modulated radiotherapy" OR "cancer complications" OR "Sjogren's Syndrome" OR "Sjogrens Syndrome" OR "Syndrome, Sjogren's" OR "Sjogren Syndrome" OR "Sicca Syndrome" OR "Syndrome, Sicca" OR "Diabetes Mellitus" OR "Diabetes Insipidus" OR "Diet, Diabetic" OR "Prediabetic State" OR "Scleredema Adultorum" OR "Glycation End Products, Advanced" OR "Glucose Intolerance" OR "Gastroparesis" OR "Antihypertensive Agents" OR "Agents, Antihypertensive" OR "Antihypertensive Agent" OR "Agent, Antihypertensive" OR "Anti-Hypertensive Agent" OR "Agent, Anti-Hypertensive" OR "Anti Hypertensive Agent" OR "Anti-Hypertensive Drug" OR "Anti Hypertensive Drug" OR "Drug, Anti-Hypertensive" OR "Antihypertensive Drug" OR "Drug, Antihypertensive" OR "Antihypertensives" OR "Anti-Hypertensive Agents" OR "Agents, Anti-Hypertensive" OR "Anti Hypertensive Agents" OR "Anti-Hypertensive Drugs" OR "Anti Hypertensive Drugs" OR "Drugs, Anti-Hypertensive" OR "Anti-Hypertensives" OR "Anti Hypertensives" OR "Antihypertensive Drugs" OR "Drugs, Antihypertensive" OR "Anti-Hypertensive" OR "Anti Hypertensive" OR "Antihypertensive" OR "Cystic Fibrosis" OR "Fibrosis, Cystic" OR "Mucoviscidosis" OR "Pulmonary Cystic Fibrosis" OR "Cystic Fibrosis, Pulmonary" OR "Pancreatic Cystic Fibrosis" OR "Cystic Fibrosis, Pancreatic" OR "Fibrocystic Disease of Pancreas" OR "Pancreas Fibrocystic Disease" OR "Pancreas Fibrocystic Diseases" OR "Cystic Fibrosis of Pancreas" OR "Cholinergic Antagonists" OR "Antagonists, Cholinergic" OR "Cholinergic Receptor Antagonist" OR "Antagonist, Cholinergic Receptor" OR "Receptor Antagonist, Cholinergic" OR "Cholinergic-Blocking Agent" OR "Agent, Cholinergic-Blocking" OR "Cholinergic Blocking Agent" OR "Cholinolytic" OR "Cholinolytics" OR "Acetylcholine Antagonist" OR "Antagonist, Acetylcholine" OR "Cholinergic Antagonist" OR "Antagonist, Cholinergic" OR "Cholinergic Receptor Antagonists" OR "Antagonists, Cholinergic Receptor" OR "Receptor Antagonists, Cholinergic" OR "Cholinergic-Blocking Agents" OR "Agents, Cholinergic-Blocking" OR "Cholinergic Blocking Agents" OR "Acetylcholine Antagonists" OR "Antagonists, Acetylcholine" OR "Anticholinergic Agents" OR "Agents, Anticholinergic" OR "Anticholinergic Agent" OR "Agent, Anticholinergic" OR "Anticholinergics" OR "Anti-Cholinergics" OR "Anti Cholinergics" OR "Anti-Cholinergic" OR "Anti Cholinergic" OR "Anticholinergic" OR "Histamine Antagonists" OR "Antagonists, Histamine" OR "Antihistamines" OR "Histamine Antagonist" OR "Antagonist, Histamine" OR "Antihistamine" OR "Antidepressive Agents" OR "Agents, Antidepressive" OR "Antidepressive Agent" OR "Agent, Antidepressive" OR "Antidepressants" OR "Antidepressant" OR "Antidepressant Drugs" OR "Drugs, Antidepressant" OR "Antidepressant Drug" OR "Drug, Antidepressant" OR "Thymoleptics" OR "Thymoleptic" OR "Thymoanaleptics" OR "Thymoanaleptic" OR "Antiparkinson Agents" OR "Agents, Antiparkinson" OR "Antiparkinsonian Agents" OR "Agents, Antiparkinsonian" OR "Antiparkinsonians" OR "Antiparkinson Drugs" OR "Drugs, Antiparkinson" OR "Anti-Anxiety Agents" OR "Agents, Anti-Anxiety" OR "Anti Anxiety Agents" OR "Anti-Anxiety Drug" OR "Anti Anxiety Drug" OR "Drug, Anti-Anxiety" OR "Anti-Anxiety Drugs" OR "Anti Anxiety Drugs" OR "Drugs, Anti-Anxiety" OR "Anxiolytic" OR "Anxiolytics" OR "Tranquilizing Agents, Minor" OR "Agents, Minor Tranquilizing" OR "Minor Tranquilizing Agents" OR	439

	"Tranquillizing Agents, Minor" OR "Agents, Minor Tranquillizing" OR "Minor Tranquillizing Agents" OR "Anxiolytic Agent" OR "Agent, Anxiolytic" OR "Anti-Anxiety Agent" OR "Agent, Anti-Anxiety" OR "Anti Anxiety Agent" OR "Anxiolytic Agents" OR "Agents, Anxiolytic" OR "Anti-Anxiety Effect" OR "Anti Anxiety Effect" OR "Effect, Anti-Anxiety" OR "Anxiolytic Effects" OR "Effects, Anxiolytic" OR "Antianxiety Effects" OR "Effects, Antianxiety" OR "Anxiolytic Effect" OR "Effect, Anxiolytic" OR "Anti-Anxiety Effects" OR "Anti Anxiety Effects" OR "Effects, Anti-Anxiety" OR "Antianxiety Effect" OR "Effect, Antianxiety" OR "Obesity" OR "xerostomia" OR "Xerostomias" OR "xerostomy" OR "Hyposalivation" OR "Hyposalivations" OR "Asialia" OR "Salivary gland dysfunction" OR "Mouth Dryness" OR "Dryness, Mouth" OR "oral dryness" OR "Salivary hypo-function" OR "dry mouth" OR "Radiation-induced xerostomia") AND ("Transcutaneous Electric Nerve Stimulation" OR "Electric Stimulation, Transcutaneous" OR "Stimulation, Transcutaneous Electric" OR "Transcutaneous Electric Stimulation" OR "Percutaneous Electric Nerve Stimulation" OR "TENS" OR "Electrical Stimulation, Transcutaneous" OR "Transcutaneous Electrical Stimulation" OR "Transdermal Electrostimulation" OR "Electrostimulation, Transdermal" OR "Percutaneous Electrical Nerve Stimulation" OR "Transcutaneous Electrical Nerve Stimulation" OR "Transcutaneous Nerve Stimulation" OR "Nerve Stimulation, Transcutaneous" OR "Stimulation, Transcutaneous Nerve" OR "Percutaneous Neuromodulation Therapy" OR "Neuromodulation Therapy, Percutaneous" OR "Percutaneous Neuromodulation Therapies" OR "Therapy, Percutaneous Neuromodulation" OR "Percutaneous Electrical Neuromodulation" OR "Electrical Neuromodulation, Percutaneous" OR "Electrical Neuromodulations, Percutaneous" OR "Neuromodulation, Percutaneous Electrical" OR "Neuromodulations, Percutaneous Electrical" OR "Percutaneous Electrical Neuromodulations" OR "Chewing Gums" OR "Chewing Gum" OR "Gum, Chewing" OR "Gums, Chewing" OR "Chewing" OR "Low-Level Light Therapy" OR "Light Therapies, Low-Level" OR "Light Therapy, Low-Level" OR "Low Level Light Therapy" OR "Low-Level Light Therapies" OR "Therapies, Low-Level Light" OR "Therapy, Low-Level Light" OR "Photobiomodulation Therapy" OR "Photobiomodulation Therapies" OR "Therapies, Photobiomodulation" OR "Therapy, Photobiomodulation" OR "LLLT" OR "Laser Therapy, Low-Level" OR "Laser Therapies, Low-Level" OR "Laser Therapy, Low Level" OR "Low-Level Laser Therapies" OR "Laser Irradiation, Low-Power" OR "Irradiation, Low-Power Laser" OR "Laser Irradiation, Low Power" OR "Low-Power Laser Therapy" OR "Low Power Laser Therapy" OR "Laser Therapy, Low-Power" OR "Laser Therapies, Low-Power" OR "Laser Therapy, Low Power" OR "Low-Power Laser Therapies" OR "Low-Level Laser Therapy" OR "Low Level Laser Therapy" OR "Low-Power Laser Irradiation" OR "Low Power Laser Irradiation" OR "Laser Biostimulation" OR "Biostimulation, Laser" OR "Laser Phototherapy" OR "Phototherapy, Laser" OR "Acupuncture" OR "Pharmacopuncture" OR "acupuncture therapy" OR "Acupuncture Treatment" OR "Acupuncture Treatments" OR "Treatment, Acupuncture" OR "Therapy, Acupuncture" OR "Pharmacoacupuncture Treatment" OR "Treatment, Pharmacoacupuncture" OR "Pharmacoacupuncture Therapy" OR "Therapy, Pharmacoacupuncture" OR "Acupotomy" OR "Acupotomies") AND ("Increased Salivary Flow" OR "Increased salivary volume" OR "Salivary flow" OR "saliva amount" OR "saliva quantity" OR "sialometry" OR "salivary flow rate" OR "salivary glands" OR "sialogogue" OR "salivary stimulations" OR "salivations" OR "parotid salivary flow" OR "unstimulated saliva flow" OR "salivary function" OR "secretory rate" OR "sublingual gland" OR "submandibular gland"))	
Web of Science	("head and neck neoplasms" OR "Neoplasms, Head and Neck" OR "head-and-neck cancer" OR "Head, Neck Neoplasms" OR "Head and Neck Neoplasm" OR "Cancer of Head and Neck" OR "Head and Neck Cancer" OR "Cancer of the Head and Neck" OR "Head Neoplasms" OR "Neoplasms, Head" OR "Head Neoplasm" OR "Neoplasm, Head" OR "Neck Neoplasms" OR "Neoplasms, Neck" OR "Neck Neoplasm" OR "Neoplasm, Neck" OR "Cancer of Head" OR "Head Cancers" OR "Head Cancer" OR "Cancer, Head" OR "Cancers, Head" OR "Cancer of the Head" OR "Cancer of Neck" OR "Neck Cancers" OR "Neck Cancer" OR "Cancer, Neck" OR "Cancers, Neck" OR "Cancer of the Neck" OR "radiotherapy" OR "Radiotherapies" OR "Radiation Therapy" OR "intensity-modulated radiotherapy" OR "Radiation Therapies" OR "Therapies, Radiation" OR "Therapy, Radiation" OR "Radiation Treatment" OR "Radiation Treatments" OR "Treatment, Radiation" OR "Radiotherapy, Targeted" OR "Radiotherapies, Targeted" OR "Targeted Radiotherapies" OR "Targeted Radiotherapy" OR "Targeted Radiation Therapy" OR "Radiation Therapies, Targeted" OR "Radiation Dosage" OR "Iodine Radioisotopes" OR "Targeted Radiation Therapies" OR "Therapies, Targeted Radiation" OR "Therapy, Targeted Radiation" OR "Radiation Therapy, Targeted" OR "Radiation Injuries" OR "radiation effects" OR "intensity-modulated radiotherapy" OR "cancer complications" OR "Sjogren's Syndrome" OR "Sjogrens Syndrome" OR "Syndrome, Sjogren's" OR "Sjogren Syndrome" OR "Sicca Syndrome" OR "Syndrome, Sicca" OR "Diabetes Mellitus" OR "Diabetes Insipidus" OR "Diet, Diabetic" OR "Prediabetic State" OR "Scleredema Adultorum" OR "Glycation End Products, Advanced" OR "Glucose Intolerance" OR "Gastroparesis" OR "Antihypertensive Agents" OR "Agents, Antihypertensive" OR "Antihypertensive Agent" OR "Agent, Antihypertensive" OR "Anti-Hypertensive Agent" OR "Agent, Anti-Hypertensive" OR "Anti Hypertensive Agent" OR "Anti-Hypertensive Drug" OR "Anti Hypertensive Drug" OR "Drug, Anti-Hypertensive" OR "Antihypertensive Drug" OR "Drug, Antihypertensive" OR "Antihypertensives" OR "Anti-Hypertensive Agents" OR "Agents, Anti-Hypertensive" OR "Anti Hypertensive Agents" OR "Anti-Hypertensive Drugs" OR "Anti Hypertensive Drugs" OR "Drugs, Anti-Hypertensive" OR "Anti-Hypertensives" OR "Anti Hypertensives" OR "Antihypertensive Drugs" OR "Drugs, Antihypertensive" OR "Anti-Hypertensive" OR "Anti Hypertensive" OR "Antihypertensive" OR "Cystic Fibrosis" OR "Fibrosis, Cystic" OR "Mucoviscidosis" OR "Pulmonary Cystic Fibrosis" OR "Cystic Fibrosis, Pulmonary" OR "Pancreatic Cystic Fibrosis" OR "Cystic Fibrosis, Pancreatic" OR "Fibrocystic Disease of Pancreas" OR "Pancreas Fibrocystic Disease" OR "Pancreas Fibrocystic Diseases" OR "Cystic Fibrosis of Pancreas" OR "Cholinergic Antagonists" OR "Antagonists, Cholinergic" OR "Cholinergic Receptor Antagonist" OR "Antagonist, Cholinergic Receptor" OR "Receptor Antagonist, Cholinergic" OR "Cholinergic-Blocking Agent" OR "Agent, Cholinergic-Blocking" OR "Cholinergic	245

	Blocking Agent" OR "Cholinolytic" OR "Cholinolytics" OR "Acetylcholine Antagonist" OR "Antagonist, Acetylcholine" OR "Cholinergic Antagonist" OR "Antagonist, Cholinergic" OR "Cholinergic Receptor Antagonists" OR "Antagonists, Cholinergic Receptor" OR "Receptor Antagonists, Cholinergic" OR "Cholinergic-Blocking Agents" OR "Agents, Cholinergic-Blocking" OR "Cholinergic Blocking Agents" OR "Acetylcholine Antagonists" OR "Antagonists, Acetylcholine" OR "Anticholinergic Agents" OR "Agents, Anticholinergic" OR "Anticholinergic Agent" OR "Agent, Anticholinergic" OR "Anticholinergics" OR "Anti-Cholinergics" OR "Anti Cholinergics" OR "Anti-Cholinergic" OR "Anti Cholinergic" OR "Anticholinergic" OR "Histamine Antagonists" OR "Antagonists, Histamine" OR "Antihistamines" OR "Histamine Antagonist" OR "Antagonist, Histamine" OR "Antihistamine" OR "Antidepressive Agents" OR "Agents, Antidepressive" OR "Antidepressive Agent" OR "Agent, Antidepressive" OR "Antidepressants" OR "Antidepressant" OR "Antidepressant Drugs" OR "Drugs, Antidepressant" OR "Antidepressant Drug" OR "Drug, Antidepressant" OR "Thymoleptics" OR "Thymoleptic" OR "Thymoanaleptics" OR "Thymoanaleptic" OR "Antiparkinson Agents" OR "Agents, Antiparkinson" OR "Antiparkinsonian Agents" OR "Agents, Antiparkinsonian" OR "Antiparkinsonians" OR "Antiparkinson Drugs" OR "Drugs, Antiparkinson" OR "Anti-Anxiety Agents" OR "Agents, Anti-Anxiety" OR "Anti Anxiety Agents" OR "Anti-Anxiety Drug" OR "Anti Anxiety Drug" OR "Drug, Anti-Anxiety" OR "Anti-Anxiety Drugs" OR "Anti Anxiety Drugs" OR "Drugs, Anti-Anxiety" OR "Anxiolytic" OR "Anxiolytics" OR "Tranquilizing Agents, Minor" OR "Agents, Minor Tranquilizing" OR "Minor Tranquilizing Agents" OR "Tranquillizing Agents, Minor" OR "Agents, Minor Tranquillizing" OR "Minor Tranquillizing Agents" OR "Anxiolytic Agent" OR "Agent, Anxiolytic" OR "Anti-Anxiety Agent" OR "Agent, Anti-Anxiety" OR "Anti Anxiety Agent" OR "Anxiolytic Agents" OR "Agents, Anxiolytic" OR "Anti-Anxiety Effect" OR "Anti Anxiety Effect" OR "Effect, Anti-Anxiety" OR "Anxiolytic Effects" OR "Effects, Anxiolytic" OR "Antianxiety Effects" OR "Effects, Antianxiety" OR "Anxiolytic Effect" OR "Effect, Anxiolytic" OR "Anti-Anxiety Effects" OR "Anti Anxiety Effects" OR "Effects, Anti-Anxiety" OR "Antianxiety Effect" OR "Effect, Antianxiety" OR "Obesity" OR "xerostomia" OR "Xerostomias" OR "xerostomy" OR "Hyposalivation" OR "Hyposalivations" OR "Asialia" OR "Salivary gland dysfunction" OR "Mouth Dryness" OR "Dryness, Mouth" OR "oral dryness" OR "Salivary hypo-function" OR "dry mouth" OR "Radiation-induced xerostomia") AND ("Transcutaneous Electric Nerve Stimulation" OR "Electric Stimulation, Transcutaneous" OR "Stimulation, Transcutaneous Electric" OR "Transcutaneous Electric Stimulation" OR "Percutaneous Electric Nerve Stimulation" OR "TENS" OR "Electrical Stimulation, Transcutaneous" OR "Transcutaneous Electrical Stimulation" OR "Transdermal Electrostimulation" OR "Electrostimulation, Transdermal" OR "Percutaneous Electrical Nerve Stimulation" OR "Transcutaneous Electrical Nerve Stimulation" OR "Transcutaneous Nerve Stimulation" OR "Nerve Stimulation, Transcutaneous" OR "Stimulation, Transcutaneous Nerve" OR "Percutaneous Neuromodulation Therapy" OR "Neuromodulation Therapy, Percutaneous" OR "Percutaneous Neuromodulation Therapies" OR "Therapy, Percutaneous Neuromodulation" OR "Percutaneous Electrical Neuromodulation" OR "Electrical Neuromodulation, Percutaneous" OR "Electrical Neuromodulations, Percutaneous" OR "Neuromodulation, Percutaneous Electrical" OR "Neuromodulations, Percutaneous Electrical" OR "Percutaneous Electrical Neuromodulations" OR "Chewing Gums" OR "Chewing Gum" OR "Gum, Chewing" OR "Gums, Chewing" OR "Chewing" OR "Low-Level Light Therapy" OR "Light Therapies, Low-Level" OR "Light Therapy, Low-Level" OR "Low Level Light Therapy" OR "Low-Level Light Therapies" OR "Therapies, Low-Level Light" OR "Therapy, Low-Level Light" OR "Photobiomodulation Therapy" OR "Photobiomodulation Therapies" OR "Therapies, Photobiomodulation" OR "Therapy, Photobiomodulation" OR "LLLT" OR "Laser Therapy, Low-Level" OR "Laser Therapies, Low-Level" OR "Laser Therapy, Low Level" OR "Low-Level Laser Therapies" OR "Laser Irradiation, Low-Power" OR "Irradiation, Low-Power Laser" OR "Laser Irradiation, Low Power" OR "Low-Power Laser Therapy" OR "Low Power Laser Therapy" OR "Laser Therapy, Low-Power" OR "Laser Therapies, Low-Power" OR "Laser Therapy, Low Power" OR "Low-Power Laser Therapies" OR "Low-Level Laser Therapy" OR "Low Level Laser Therapy" OR "Low-Power Laser Irradiation" OR "Low Power Laser Irradiation" OR "Laser Biostimulation" OR "Biostimulation, Laser" OR "Laser Phototherapy" OR "Phototherapy, Laser" OR "Acupuncture" OR "Pharmacopuncture" OR "acupuncture therapy" OR "Acupuncture Treatment" OR "Acupuncture Treatments" OR "Treatment, Acupuncture" OR "Therapy, Acupuncture" OR "Pharmacoacupuncture Treatment" OR "Treatment, Pharmacoacupuncture" OR "Pharmacoacupuncture Therapy" OR "Therapy, Pharmacoacupuncture" OR "Acupotomy" OR "Acupotomies") AND ("Increased Salivary Flow" OR "Increased salivary volume" OR "Salivary flow" OR "saliva amount" OR "saliva quantity" OR "sialometry" OR "salivary flow rate" OR "salivary glands" OR "sialogogue" OR "salivary stimulations" OR "salivations" OR "parotid salivary flow" OR "unstimulated saliva flow" OR "salivary function" OR "secretory rate" OR "sublingual gland" OR "submandibular gland")	
Google Scholar Web Search	("Transcutaneous Electric Nerve Stimulation" OR "Low Power Laser Therapy" OR "Acupuncture Therapy" OR "Chewing Gum") AND ("radiotherapy" OR "Sjogren's Syndrome" OR "Diabetes Mellitus" OR "Antihypertensive Agents") AND ("salivary flow") AND ("hyposalivation")	100
Cochrane	("head and neck neoplasms" OR "head-and-neck cancer" OR "Head, Neck Neoplasms" OR "Head and Neck Neoplasm" OR "Cancer of Head and Neck" OR "Head and Neck Cancer" OR "Cancer of the Head and Neck" OR "Head Neoplasms" OR "Head Neoplasm" OR "Neck Neoplasms" OR "Neck Neoplasm" OR "Cancer of Head" OR "Head Cancers" OR "Head Cancer" OR "Cancer of the Head" OR "Cancer of Neck" OR "Neck Cancers" OR "Neck Cancer" OR "Cancer of the Neck" OR "radiotherapy" OR "Radiotherapies" OR "Radiation Therapy" OR "intensity-modulated radiotherapy" OR "Radiation Therapies" OR "Radiation Treatment" OR "Radiation Treatments" OR "Targeted Radiotherapies" OR "Targeted Radiotherapy" OR "Targeted Radiation Therapy" OR "Radiation Dosage" OR "Iodine	117

	Radioisotopes" OR "Targeted Radiation Therapies" OR "Radiation Injuries" OR "radiation effects" OR "intensity-modulated radiotherapy" OR "cancer complications" OR "Sjogren's Syndrome" OR "Sjogrens Syndrome" OR "Sjogren Syndrome" OR "Sicca Syndrome" OR "Diabetes Mellitus" OR "Diabetes Insipidus" OR "Prediabetic State" OR "Scleredema Adultorum" OR "Glucose Intolerance" OR "Gastroparesis" OR "Antihypertensive Agents" OR "Antihypertensive Agent" OR "Anti-Hypertensive Agent" OR "Anti Hypertensive Agent" OR "Anti-Hypertensive Drug" OR "Anti Hypertensive Drug" OR "Antihypertensive Drug" OR "Antihypertensives" OR "Anti-Hypertensive Agents" OR "Anti Hypertensive Agents" OR "Anti-Hypertensive Drugs" OR "Anti Hypertensive Drugs" OR "Anti-Hypertensives" OR "Anti Hypertensives" OR "Antihypertensive Drugs" OR "Anti-Hypertensive" OR "Anti Hypertensive" OR "Antihypertensive" OR "Cystic Fibrosis" OR "Mucoviscidosis" OR "Pulmonary Cystic Fibrosis" OR "Pancreatic Cystic Fibrosis" OR "Fibrocystic Disease of Pancreas" OR "Pancreas Fibrocystic Disease" OR "Pancreas Fibrocystic Diseases" OR "Cystic Fibrosis of Pancreas" OR "Cholinergic Antagonists" OR "Cholinergic Receptor Antagonist" OR "Cholinergic-Blocking Agent" OR "Cholinergic Blocking Agent" OR "Cholinolytic" OR "Cholinolytics" OR "Acetylcholine Antagonist" OR "Cholinergic Antagonist" OR "Cholinergic Receptor Antagonists" OR "Cholinergic-Blocking Agents" OR "Cholinergic Blocking Agents" OR "Acetylcholine Antagonists" OR "Anticholinergic Agents" OR "Anticholinergic Agent" OR "Anticholinergics" OR "Anti-Cholinergics" OR "Anti Cholinergics" OR "Anti-Cholinergic" OR "Anti Cholinergic" OR "Anticholinergic" OR "Histamine Antagonists" OR "Antihistamines" OR "Histamine Antagonist" OR "Antihistamine" OR "Antidepressive Agents" OR "Antidepressive Agent" OR "Antidepressants" OR "Antidepressant" OR "Antidepressant Drugs" OR "Antidepressant Drug" OR "Thymoleptics" OR "Thymoleptic" OR "Thymoanaleptics" OR "Thymoanaleptic" OR "Antiparkinson Agents" OR "Antiparkinsonian Agents" OR "Antiparkinsonians" OR "Antiparkinson Drugs" OR "Anti-Anxiety Agents" OR "Anti Anxiety Agents" OR "Anti-Anxiety Drug" OR "Anti Anxiety Drug" OR "Anti-Anxiety Drugs" OR "Anti Anxiety Drugs" OR "Anxiolytic" OR "Anxiolytics" OR "Minor Tranquilizing Agents" OR "Minor Tranquillizing Agents" OR "Anxiolytic Agent" OR "Anti-Anxiety Agent" OR "Anti Anxiety Agent" OR "Anxiolytic Agents" OR "Anti-Anxiety Effect" OR "Anti Anxiety Effect" OR "Anxiolytic Effects" OR "Antianxiety Effects" OR "Anxiolytic Effect" OR "Anti-Anxiety Effects" OR "Anti Anxiety Effects" OR "Antianxiety Effect" OR "Obesity" OR "xerostomia" OR "Xerostomias" OR "xerostomy" OR "Hyposalivation" OR "Hyposalivations" OR "Asialia" OR "Salivary gland dysfunction" OR "Mouth Dryness" OR "oral dryness" OR "Salivary hypo-function" OR "dry mouth" OR "Radiation-induced xerostomia") AND ("Transcutaneous Electric Nerve Stimulation" OR "Transcutaneous Electric Stimulation" OR "Percutaneous Electric Nerve Stimulation" OR "TENS" OR "Electrical Stimulation, Transcutaneous" OR "Transcutaneous Electrical Stimulation" OR "Transdermal Electrostimulation" OR "Percutaneous Electrical Nerve Stimulation" OR "Transcutaneous Electrical Nerve Stimulation" OR "Transcutaneous Nerve Stimulation" OR "Percutaneous Neuromodulation Therapy" OR "Percutaneous Neuromodulation Therapies" OR "Percutaneous Electrical Neuromodulation" OR "Percutaneous Electrical Neuromodulations" OR "Chewing Gums" OR "Chewing Gum" OR "Chewing" OR "Low-Level Light Therapy" OR "Low Level Light Therapy" OR "Low-Level Light Therapies" OR "Photobiomodulation Therapy" OR "Photobiomodulation Therapies" OR "LLLT" OR "Low-Level Laser Therapies" OR "Low-Power Laser Therapy" OR "Low Power Laser Therapy" OR "Low-Power Laser Therapies" OR "Low-Level Laser Therapy" OR "Low Level Laser Therapy" OR "Low-Power Laser Irradiation" OR "Low Power Laser Irradiation" OR "Laser Biostimulation" OR "Laser Phototherapy" OR "Acupuncture" OR "Pharmacopuncture" OR "acupuncture therapy" OR "Acupuncture Treatment" OR "Acupuncture Treatments" OR "Pharmacoacupuncture Treatment" OR "Pharmacoacupuncture Therapy" OR "Acupotomy" OR "Acupotomies") AND ("Increased Salivary Flow" OR "Increased salivary volume" OR "Salivary flow" OR "saliva amount" OR "saliva quantity" OR "sialometry" OR "salivary flow rate" OR "salivary glands" OR "sialogogue" OR "salivary stimulations" OR "salivations" OR "parotid salivary flow" OR "unstimulated saliva flow" OR "salivary function" OR "secretory rate" OR "sublingual gland" OR "submandibular gland")	

Appendix table 2. Excluded studies and reasons for exclusion

Author, year	Reasons for exclusion *
Aagaard et al., 1992	4
Aguiar et al., 2021	3
Aparna et al., 2017	7
Barbosa et al., 2018	8
Blom et al., 1993	1
Blom et al., 1993	6
Blom et al., 1996	10
Blom et al., 1999	6
Bokkasam et al., 2020	10
Bots et al., 2004	4
Bots et al., 2005	2
Bots et al., 2005	4
Braga et al., 2010	2
Braga et al., 2011	10
Bussadori, 2018	3
Chang et al., 2021	8
Chhugani et al., 2021	7
Cho et al., 2008	10
Dakovic et al., 2021	8
Dantas et al., 2020	10
Davies, 2018 / 3	3
Dawidson et al., 1998	1
Dawidson et al., 1998	1
Dawidson et al., 1998	8
Dawidson et al., 1999	1
Duruk et al., 2016	4
Dutta et al., 2021	2
Dutta et al., 2021	2
Fedele, 2011	3
Frost et al., 2006	4
Garcia et al., 2009	10
Garcia et al., 2019	1

Gonçalves et al., 2020	3
Gonnelli et al., 2016	5
Gonnelli et al., 2016	5
Gueimonde et al., 2016	4
Haghighatafshar, 2014	3
Hakuta et al., 2009	5
Huang, 2020	3
Iovoli et al., 2020	1
Ismail, 2020	3
Jagodzińska et al., 2011	4
Jensen et al., 1991	6
Jiang et al., 2017	1
Johnson, 2020	3
Jung et al., 2020	8
Juras et al., 2010	7
Kaae et al., 2015	2
Kaae et al., 2016	4
Kaae et al., 2020	4
Kim et al., 2021	4
Leite et al., 2012	2
Libik et al., 2017	10
List et al., 1998	5
Lončar et al., 2011	7
Lopes et al., 2006	10
Louzeiro et al., 2020	8
Lu et al., 2012	1
Mandel, 2012	4
Martins et al., 2019	1
Meneguzzo et al., 2021	2
Meng et al., 2012	10
Meng et al., 2012	10
Mirjalili et al., 2012	3
Mirjalili et al., 2013	2
Mohammed, 2013	4

Morganstein, 2005	6
Motta, 2019	3
Narmatha et al., 2020	2
Nemeth et al., 2020	4
O'Gorman et al., 2016	2
Ojha et al., 2016	10
Oliveira et al., 2020	9
Olsson et al., 1991	4
Olsson et al., 1991	4
Österberg et al., 1992	5
Oton-Leite et al., 2013	5
Ozen et al., 2020	4
Paim et al., 2018	6
Paim et al., 2019	10
Pavesi et al., 2016	2
Pavesi et al., 2021	8
Pillay, 2014	4
Prinsloo et al., 2018	2
Raber-Durlacher et al., 2019	2
Rangare et al., 2015	10
Ribeiro et al., 2021	5
Risheim et al., 1993	4
Saleh et al., 2014	8
Saleh, 2014	2
Sayáns, 2021	3
Sholikhah et al., 2020	4
Simcock et al., 2009	10
Simcock et al., 2010	2
Simcock et al., 2013	10
Simões et al., 2010	8
Simons et al., 2001	4
Sjögren et al., 1993	4
Stewart et al., 1998	6
Varellis et al., 2020	7

Wibawa et al., 2018	5
Wolff et al., 2018	6
Wong et al., 2003	10
Wong et al., 2012	6
Wong et al., 2015	10
Wyatt et al., 2016	1
Yang et al., 2010	8
Yang et al., 2019	8
Yousef, 2021	3
Zukauskaite et al., 2015	2

1. Clinical results without quantitative analysis of the salivary flow
2. Not original research or conference abstracts
3. Study protocol
4. Only chewing gum or other non-physical method
5. Presence of prosthesis wearers
6. Presence of confounding bias (sialogogues and salivary substitutes)
7. unknown cause for hyposalivation
8. No distinguished results for different groups
9. Pediatric participants
10. Irradiated participants

Appendix table 3. Data extraction table containing the characteristics and results of included studies (N = 24). NA = Not available; * = represents the same study, but test groups and their results were divided by different causes of hyposalivation; SIG = Statistically significant; NS = Statistically not significant; RCT = Randomized clinical trial; NRCT= Non-randomized clinical trial; SACT = Single-arm clinical trial; CR = Case report; USF = Unstimulated salivary flow; SSF= Stimulated salivary flow.

Author, country, year, type of study	physical method	Test(s) group(s)<br><br>N and age	Control(s) group(s)<br><br>N and age	Protocol	Sialometry (collection times and types)	Salivary flow Before (mean ± SD)	Salivary flow After (mean ± SD)	Outcomes
Studies in individuals with Sjögren's Syndrome								
Blom et al. *<br><br>Switzerland<br><br>1992<br><br>RCT	Acupuncture	N G1 = 11 (Acupuncture)<br><br>Age G1 (mean) = 58 years<br><br>(In this case, only patient U, from G2).	N G2 = 10 (Superficial acupuncture)<br><br>Age G2 (mean) = 62 years	G1 = 6 to 8 local and distal points and 2 to 4 auricular points, with insertions at depths between 0.5 and 2.0 cm and approximately 1 to 3 mm into the ear.<br><br>G2 = intraoperative needles dermal (superficial acupuncture).<br><br>G1 and G2 = 2x/week, 20 minutes, for 6 weeks. Interval of 7 to 10 days and protocol repetition for another 6 weeks.	Saliva collection unstimulated and stimulated by paraffin at two different times before starting treatment.<br><br>Samples were collected twice before the second series of acupuncture treatments.<br><br>New salivary assessments were made immediately after the completion of the acupuncture series; in 3 weeks; and 3, 6 and 12 months after the end of treatment.	USF = 0.01 mL/min<br><br>SSF = 0.22 mL/min	Immediately after:<br>USF = 0.12 mL/min<br><br>SSF = 0.22 mL/min<br><br>12 months later:<br>USF = 0.06 mL/min<br><br>SSF = 0.20 mL/min	G1 showed improved salivary flow rates during and after treatment. 4 out of 11 patients achieved normal salivary flow rates. The results persisted after 12 months.<br><br>G2 showed improvement in their salivary flow rates during treatment, but these changes disappeared after completing the protocol.<br><br>Patients using mechanical stimulants and salivary substitutes were excluded.
Cafaro et al.<br><br>Italy<br><br>2014<br><br>RCT	Laser acupuncture	N G1 = 14 (laser acupuncture)<br><br>Age (mean ± SD) = 69,31 years (±9,83)	N G2 = 12 (placebo)<br><br>Age (mean ± SD) = 69,31 years (±9,83)	G1 = Light in the red visible spectrum (650nm), power of 5 mW, 120 seconds per acupuncture point, total dose of 0.6J.<br><br>1x/week for 5 weeks (5 sessions).<br>6 acupuncture points stimulated bilaterally.	Schirmer oral test (WST).<br><br>1 week before the first laser procedure (T0), after each laser session (T1-T5) and 30 (T6), 90 (T7), 180 (T8) days later.	G1 (T0) = 1.12 (±0.96)mm/min<br><br>G2 (T0)= 0.90 (±0.22)mm/min	G1 (T5) = 5.94 (±1.64)mm/min<br><br>G2 (T5) = 0.92 (±0.39)mm/min	G1 showed an increase in salivation at the end, with stability up to the 3rd month (T6) of follow-up and a reduction from the 3rd to the 6th month (T8).<br><br>G1 showed a greater SIG increase than G2 at the end of the protocol (T5).

				G2 = G1 protocol, but without emitting radiation.			G1 (T6) = 5.81 (±7.60)mm/min<br><br>G2 (T6) = 0.98 (±0.29)mm/min<br><br>G1 (T8)= 4.69 (±5.96)mm/min<br><br>G2 (T8) = 0.91 (±0.34)mm/min	
Fidelix et al.<br><br>Brazil<br><br>2018<br><br>RCT	Laser	N G1 = 33 (Laser)<br><br>Age G1 (mean ± SD) = 53.9 years (49.9–57.9)	N G2 = 33 (Placebo)<br><br>Age G2 (mean ± SD) = 57.2 years (53.3–61.2)	G1 = Wavelength 808 nm, 100 mW, and in continuous wave mode.<br><br>2x/week for 6 weeks (12 sessions).<br><br>12 extraoral irradiation points and 2 intraoral irradiation points.<br><br>4 J per stitch for 40 seconds. Total energy dose per session: 56 J.<br><br>G2 = G1 protocol, but without emitting radiation.	At rest, 2% citric acid was applied to the dorsum of the tongue. For 10 minutes, participants were asked to spit out whole saliva.<br><br>Collection performed before and after treatment.	G1 = 0.122 (0.082–0.163) mL/min<br><br>G2 = 0.170 (0.102–0.238) mL/min	G1 = 0.136 (0.096–0.176) mL/min<br><br>G2 = 0.172 (0.114–0.230) mL/min	There was no difference within groups or between groups.
Simões et al.<br><br>Brazil<br><br>2009<br><br>CR	Laser	N = 1<br><br>60 years		G1 = Wavelength of 780nm, power of 15mW, in continuous wave mode, dose of 3.8J/cm².<br><br>For 8 months, treatment was given 3x/week for 4 weeks, followed by a 1-week break, and then treatment was	Total stimulated saliva was collected after chewing parafilm before and during the laser for 30 minutes.	Stimulated total saliva: 0,033 ml/min	Stimulated total saliva: 0,036ml/min	Salivary flow rate did not increase by more than 10%.

				resumed.<br><br>10 seconds per point. 30 extraoral irradiation points and 6 intraoral points.				
Studies in individuals with Diabetes mellitus								
Chandra et al. *<br><br>India<br><br>2022<br><br>NRCT	TENS	N G1 = 4 (diabetics)<br><br>N G2 = 25 (health)<br><br>Age = between 18 and 70 years		Electrodes bilaterally on the skin overlying the parotid gland region.<br><br>Frequency between 20-50 Hz, intensity according to the patient's tolerance.<br><br>2x/week every 15 days, for 1 month (4 times in total).	Unstimulated saliva ("forced spit" method) was collected for 5 minutes before TENS application.<br><br>After reaching the maximum tolerated intensity, stimulated saliva was collected for 5 minutes.<br><br>The difference between salivary flow after and before TENS application in the 4 sessions was calculated.	Mean difference in salivary flow at the 1st session:<br><br>G1 = 0.11(± 0.11)mL/min<br><br>G2 = 0.30(±0.14) mL/min	Mean difference in salivary flow at the 4th session:<br><br>G1 = 1.12(±0.43) mL/min<br><br>G2 = 1.56(±0.28) mL/min	Increased salivary flow in G1 after treatment.<br><br>G2 showed a greater SIG response than G1 after treatment.<br><br>Diabetic patients had a better response than the other groups with hyposalivation included in the study.
Dyasnoor et al.<br><br>India<br><br>2017<br><br>SACT	TENS	N = 40 (N =15 with hyposalivation)<br><br>Age (mean) = 48.15 years		Electrodes bilaterally on the skin overlying the parotid gland region.<br><br>Pulse rate fixed at 50Hz and intensity according to patient tolerance.<br><br>Single application.	Unstimulated saliva was collected ("forced spit" method) 10 minutes before TENS application.<br><br>After activating TENS, saliva was collected continuously for 10 minutes. After an interval of 1 minute, this procedure was repeated in burst mode.	Total subjects: 1.69 (± 0.33) mL/10 min<br><br>Subjects with hyposalivation:<br>1.34 (± 0.23) mL/10 min	Total subjects: 1.88 (± 0.36) mL/10 min<br><br>Subjects with hyposalivation:<br>1.55 (±0.31) mL/10 min	Increase in total saliva after application of TENS in continuous mode.<br><br>No control group to compare findings.
Shetawy et al.<br><br>Egypt<br><br>2021	Laser	N = 25<br><br>Age (mean ± SD) = 49.08 (±4.37) years		905nm wavelength, 100mW power, 2J energy per point and 28J dose per session.<br><br>2x/week for 6 weeks (total of	Unstimulated saliva was collected ("forced spit" method) for 5 minutes before laser application.	0.11 (±0.04)mL/min	0.35(±0.06) mL/min (after the 12th session)	Increased salivary flow after treatment.<br><br>No control group to compare findings.

SACT				12 sessions).<br><br>14 points of extraoral irradiation.<br>The application time was 30 seconds per point (total duration of 420 seconds).	Salivary flow was measured before the beginning of the sessions, after the 6th session and after the 12th session.			
Smriti. B et al. *<br><br>India<br><br>2014<br><br>SACT	TENS	N = 6<br><br>Age (mean) = NA		Electrodes bilaterally on the skin overlying the parotid gland region.<br><br>Pulse rate of 50 Hz, duration of 250 microseconds, unit in normal mode up to the maximum intensity tolerated by the patient.<br><br>Single application.	Unstimulated saliva was collected for 5 minutes in a beaker and measured in ml. After applying TENS for 5 minutes, stimulated saliva was collected for another 5 minutes.<br><br>The collections were performed before and after the application of TENS.	2.53 (±0.78)mL/ 5min	3.33 (±0.83) mL/5min	Increased salivary flow after TENS application.<br><br>No control group to compare findings.
Studies in postmenopausal individuals								
Blom, Dawidson and Angmar-Månsson et al. *<br><br>Switzerland<br><br>1992<br><br>RCT	Acupuncture	N G1 = 11 (Acupuncture)<br><br>Age G1 (mean) = 58 years<br><br>N G2 = 10 (Superficial acupuncturel)<br><br>Age G2 (mean) = 62 years<br><br>(In this case, only patient I, from G1).		G1 = 6 to 8 local and distal points and 2 to 4 auricular points, with insertions at depths between 0.5 and 2.0 cm and approximately 1 to 3 mm into the ear.<br><br>G2 = intraoperative needles dermal (superficial acupuncture).<br><br>G1 and G2 = 2x/week, 20 minutes, for 6 weeks. Interval of 7 to 10 days and protocol repetition for another 6 weeks.	Saliva collection unstimulated and stimulated by paraffin at two different times before starting treatment.<br><br>Samples were collected twice before the second series of acupuncture treatments.<br><br>New salivary assessments were made immediately after the completion of the acupuncture series; in 3 weeks; and 3, 6 and 12 months after the end of treatment.	USF = 0.04 mL/min<br><br>SSF= 0.14 mL/min	Immediately after:<br>USF = 0.06 mL/min<br><br>SSF= 0.10 mL/min<br><br>12 months later:<br>USF = 0.01 mL/min<br><br>SSF= 0.11 mL/min	G1 showed improved salivary flow rates during and after treatment. 4 out of 11 patients achieved normal salivary flow rates. The results persisted after 12 months.<br><br>G2 showed improvement in their salivary flow rates during treatment, but these changes disappeared after the conclusion of the protocol.<br><br>Patients using mechanical stimulants and salivary substitutes were excluded.

Konidena et al.<br><br>India<br><br>2016<br><br>NRCT	TENS	N G1 = 25 (with dry mouth)<br><br>Age G1 (mean ± SD) = 61.16 (±10.36) years<br><br>N G2 = 25 (with dry mouth)<br><br>Age G2 (mean ± SD) = 58.48 (±7.86) years		Electrodes bilaterally on the skin overlying the parotid gland region.<br><br>50 Hz frequency, 220 V, 0–100 mA at 1k load, biphasic waveform, available in pulsed/continuous form and in 2 intensities.<br><br>The TENS unit was activated in continuous mode, with maximum intensity tolerated by the patient for 15 minutes (40 patients supported intensity II);<br><br>Single application.	Unstimulated saliva was collected ("forced spit" method) for 5 minutes before TENS application.<br><br>After stimulation, saliva was collected for 5 minutes with the same method.	G1 = 0.60 (±0.61)mL/ 5 Mins<br><br>G2 = 3.40(±1.06) mL/5 Mins	G1 = 0.93 (±0,89)mL/5 Mins<br><br>G2 = 3.86 (±1,15)mL/ 5 mins	Increased salivary flow after TENS application in G1 and G2.<br><br>90% of subjects responded to TENS therapy. There was a mean increase of 0.33 (±0.27)mL in G1 and 0.46 (±0.10)mL in G2.<br><br>No control group to compare findings.
Smriti et al. *<br><br>India<br><br>2014<br><br>SACT	TENS	N = 9<br><br>Age = Over 50 years		Electrodes bilaterally on the skin overlying the parotid gland region.<br><br>Pulse rate of 50 Hz, duration of 250 microseconds, unit in normal mode up to the maximum intensity tolerated by the patient.<br><br>Single application.	Unstimulated saliva was collected for 5 minutes in a beaker and measured in ml. After applying TENS for 5 minutes, stimulated saliva was collected for another 5 minutes.<br><br>The collections were performed before and after the application of TENS.	2.14(±0.63) mL/5min	2.80(±0.70) mL/5min	Increased salivary flow after TENS application.<br><br>No control group to compare findings.
Studies in individuals using medications								
Blom et al. *<br><br>Switzerland<br><br>1992<br><br>RCT	Acupuncture	N G1 = 11 (Acupuncture)<br><br>Age G1 (mean) = 58 years<br><br>N G2 = 10 (Superficial acupuncturel)		G1 = 6 to 8 local and distal points and 2 to 4 auricular points, with insertions at depths between 0.5 and 2.0 cm and approximately 1 to 3 mm into the ear.<br><br>G2 = intraoperative needles dermal (superficial acupuncture).	Saliva collection unstimulated and stimulated by paraffin at two different times before starting treatment.<br><br>Samples were collected twice before the second series of acupuncture treatments.	USF = 0.04 mL/min<br><br>SSF = 0.34 mL/min	Immediately after:<br>USF = 0.08 mL/min<br><br>SSF = 0.40 mL/min<br><br>12 months later:<br>NA	G1 showed improved salivary flow rates during and after treatment. 4 out of 11 patients achieved normal salivary flow rates. The results persisted after 12 months.<br><br>G2 showed improvement in their salivary flow rates during treatment, but these

		Age G2 (mean) = 62 years<br><br>(In this case, only patient M, from G2).		G1 and G2 = 2x/week, 20 minutes, for 6 weeks. Interval of 7 to 10 days and protocol repetition for another 6 weeks.	New salivary assessments were made immediately after the completion of the acupuncture series; in 3 weeks; and 3, 6 and 12 months after the end of treatment.			changes disappeared after the conclusion of the protocol.<br><br>Patients using mechanical stimulants and salivary substitutes were excluded.
Brzak et al.<br><br>Croatia<br><br>2017<br><br>RCT	Laser	N G1 = 15<br><br>Age G1 (mean) = 69.53 years<br><br>N G2 = 15<br><br>Age G2 (mean)= 67.67 years		G1= Power of 35mW and wavelength of 830nm. 5.2 Hz pulse repetition rate.<br><br>G2 = Power of 30mW and wavelength of 685nm. 5.2 Hz pulse repetition rate.<br><br>Approximately 14.4J per session in G1 and G2.<br><br>6 irradiation points. Each application with different duration depending on the gland and laser wavelength.<br><br>The treatment lasted 10 consecutive days for G1 and G2.	Patients expectorated all saliva into graduated test tubes for 5 minutes.<br><br>Total amounts of unstimulated and stimulated saliva were measured before and after each laser treatment and 10 days after the last (10th) treatment.	G1 = Approximate mean value of 0.20 mL/min.<br><br>G2 = Approximate mean value between 0.15 and 0.20 mL/min.	Last day of treatment:<br><br>G1 = Approximate mean value between 0.35 e 0.40 mL/min.<br><br>G2 = Approximate mean value between 0.25 e 0.30 mL/min.<br><br>10 days after the last day:<br><br>G1 = Approximate mean value between 0.25 e 0.30 mL/min.<br><br>G2 = Approximate mean value of 0.25mL/min.	Increase in G1 and G2. Salivation remains improved 10 days after the end of treatment. G1 showed continuously higher values of saliva quantity.<br><br>No control group to compare findings.
Dabić et al.<br><br>Croatia	Laser	N G1 = 28 (Laser)	N G2 = 15 (Laser simulado)	G1 = Wavelength of 830 nm, power of 35 mW, frequency of 5.2 Hz, alternating mode	Unstimulated and stimulated salivary flow rates were measured	USF:<br>G1 = 0.12 (±0.09)mL/	USF:<br>G1 = Approximate	Increased unstimulated salivary flow in G1. The increase was NS in G2.

2016<br><br>RCT		Age (mean) = 71.3 (±7.6) years	Idade (média) = 74,0(±7,0) anos	(on: 800 ms, off: 1 ms) and dose of 1.60 J/cm².<br><br>Extraoral and intraoral irradiation points (does not inform quantity).<br><br>Each session lasted 20 min, 10 min per side of the face (total of 120 seconds of irradiation per session).<br><br>The laser was repeated every day, except on weekends, for 14 days (10 sessions).<br><br>G2 = Same protocol, but with the device turned off.	before and after the end of the laser or simulated laser.<br><br>All participants were instructed to collect saliva in their mouth for 5 minutes without swallowing and spitting into a container.	min<br><br>G2 = 0.17(± 0.10)mL/min<br><br>SSF:<br>G1 = 0.53 (±0.39)mL/min<br><br>G2 = 0.33 (±0.17)mL/min	mean value of 0.20 mL/min.<br><br>G2 = Approximate mean value of 0.21 mL/min.<br><br>SSF:<br>G1 = Approximate mean value between 0.55 and 0.60 mL/min.<br><br>G2 = Approximate mean value between 0.30 e 0.40 mL/min.	There was no significant difference in stimulated salivary flow after treatment in G1 and G2.
Dabic´ et al.<br><br>Croatia<br><br>2016<br><br>NRCT	Acupuncture	N G1 = 24 (Acupuncture)<br><br>Age G1 (mean ± SD) = 67.4 (± 9.9) years<br><br>N G2 = 23 (Water)<br><br>Age G2 (mean ± SD) = 68.0 (±8.9) years		G1 = 5 acupuncture points on both ears for 30 minutes.<br><br>Participants received press needles in one ear to be used until the second session (1 week later). The remaining three sessions were every seven days (5 sessions).<br><br>G2 = Water in a spray bottle (0.5 L) was given to participants to use ad libitum for 2 weeks. The patients did not know that it was pure water.	Unstimulated and stimulated salivary flow rates were measured before and after the end of the laser or simulated laser.<br><br>All participants were instructed to collect saliva in their mouth for 5 minutes without swallowing and spitting into a container.	Total saliva:<br>G1 = 0.10 (±0.12)mL/min<br><br>G2 = 0.11 (±0.11)mL/min	Total saliva:<br>G1 = Approximate mean value between 0.35 and 0.40 mL/min.<br><br>G2 = Approximate mean value of 0.12 mL/min.	Increase to G1 after treatment.<br>No statistically significant difference for G2.<br><br>No control group to compare findings.
Smriti et al. *<br><br>India<br><br>2014	TENS	N = 15<br><br>Age = NA		Electrodes bilaterally on the skin overlying the parotid gland region.<br><br>Pulse rate of 50 Hz, duration	Unstimulated saliva was collected for 5 minutes in a beaker and measured in ml. After applying TENS for 5 minutes,	Antipsychotics = 2.10 (±0.70)mL/5min	Antipsychotics = 2.86(±0.63) mL/5min	Increased salivary flow after TENS application.<br><br>No control group to compare findings.

SACT				of 250 microseconds, unit in normal mode up to the maximum intensity tolerated by the patient.<br><br>Single application.	stimulated saliva was collected for another 5 minutes.<br><br>The collections were performed before and after the application of TENS.	Diuretics = 2.73 (±1.23)mL/5min	Diuretics= 3.43 (±1.29)mL/5min	
Studies in subjects after chemotherapy								
Amaral et al.<br><br>Brazil<br><br>2012<br><br>RCT	TENS	N G1 = 9 (TENS)<br><br>N G2 = 8 (TENS + hyperboloid)<br><br>Age = 33.56(±12.46) years	N G3 = 8 (control)<br><br>N G4 = 10 (hyperboloid)<br><br>Age = 33.56(±12.46) years	G1 = Pulse frequency of 50 Hz and pulse duration of 250 microseconds.<br><br>3 sessions weekly for 30 minutes each.<br><br>Electrodes in the 3 regions of the face that corresponded to the major salivary glands.<br><br>G2 = TENS + Hyperboloid;<br><br>G3 = Without salivary stimulation;<br><br>G4 = Balanced masticatory exercises after meals using a hyperboloid (mechanical sialogogue), for 10 minutes, 4x a day.<br><br>Patients underwent therapy from day 7 before hematopoietic stem cell transplantation (D-7) to day 30 after transplantation (D30).	For unstimulated saliva, each patient collected all the saliva that formed in the oral cavity for 5 minutes. For stimulated saliva, patients used a hyperboloid for 5 minutes before saliva collection.<br><br>Collections performed 7 and 1 days before (D-7 and D-1) and 3 moments after transplantation (D3, D7 and D14);	Approximate average values on D-7:<br><br>USF:<br>G1 = Between 0.50 and 0.60mL/min<br><br>G2 = 0.70mL/min<br><br>G3 = Between 0.50 and 0.60mL/min<br><br>G4 = 0.65mL/min<br><br>SSF:<br>G1 = 1.75mL/min<br><br>G2 = Between 2.00 and 2.15mL/min<br><br>G3 = 1.25mL/min<br><br>G4 = Between	Approximate average values on D+14:<br><br>USF:<br>G1 = 0.55mL/min<br><br>G2 = Between 0.60 and 0.70mL/min<br><br>G3 = Between 0.30 and 0.40mL/min<br><br>G4 = 0.30mL/min<br><br>SSF:<br>G1 = Between 1.30 and 1.50mL/min<br><br>G2 = Between 1.50 and 1.75mL/min<br><br>G3 = 0.70mL/min	G1 and G2: trend towards increased unstimulated salivary flow on D+14.<br><br>G3 and G4: trend towards reduced salivary flow at rest on D+14.<br><br>No statistical difference between groups.<br><br>Groups G1 and G2 did not show reduced salivary flow rates (unstimulated and stimulated) to the same extent as groups G3 and G4.

						1.00 and 1.20mL/min	G4 = Between 0.75 and 1mL/min	
Chandra et al. *<br><br>India<br><br>2022<br><br>NRCT	TENS	N G1 = 3 (after chemotherapy)<br><br>Age = Between 18 and 70 years		Electrodes bilaterally on the skin overlying the parotid gland region.<br><br>Frequency between 20-50 Hz, intensity according to the patient's tolerance.<br><br>2x/week every 15 days, for 1 month (4 times in total).	Unstimulated saliva ("forced spit" method) was collected for 5 minutes before TENS application.<br><br>After reaching the maximum tolerated intensity, stimulated saliva was collected for 5 minutes.<br><br>The difference between salivary flow after and before TENS application in the 4 sessions was calculated.	Mean difference in salivary flow in the 1st session:<br><br>G1 = 0.02(±0.01) mL/min	Mean difference in salivary flow in the 4th session:<br><br>G1 = 0.77(±0.06) mL/min	Increased salivary flow in G1 after treatment.<br><br>G2 showed a greater response than G1 after treatment.
Studies in smokers								
Chandra et al. *<br><br>India<br><br>2022<br><br>NRCT	TENS	N G1 = 11 (smokers)<br><br>Age = between 18 and 70 years		Electrodes bilaterally on the skin overlying the parotid gland region.<br><br>Frequency between 20-50 Hz, intensity according to the patient's tolerance.<br><br>2x/week every 15 days, for 1 month (4 times in total).	Unstimulated saliva ("forced spit" method) was collected for 5 minutes before TENS application.<br><br>After reaching the maximum tolerated intensity, stimulated saliva was collected for 5 minutes.<br><br>The difference between salivary flow after and before TENS application in the 4 sessions was calculated.	Mean difference in salivary flow in the 1st session:<br><br>G1 = 0.11(±0.09) mL/min	Mean difference in salivary flow in the 4th session:<br><br>G1 = 1.11(±0.25) mL/min	Increased salivary flow in G1 after treatment.<br><br>G2 showed a greater response than G1 after treatment.
Studies in subjects after hemodialysis								

Yang et al.<br><br>China<br><br>2022<br><br>NRCT	TENS on acupuncture points	N G1 = 37<br><br>Age G1 (mean ± SD) = 59,9(±13,00) anos<br><br>N G2 = 38<br><br>Age G2 (mean ± SD) = 58.80(±11.50) years		G1 = Frequency of 50Hz and a pulse duration of 250μs. Electrodes were placed on 2 acupuncture points bilaterally and the stimulation lasted for 20 minutes.<br><br>G2 = Same treatment as G1, but with a frequency of 2Hz and a pulse of 50μs.	Measurements were taken before, immediately after the end of the 3-week treatment and 1 week after the last session.<br><br>Before the measurement, the patients swallowed their saliva and cotton rolls were inserted in their mouths for 5 min. The cotton rolls were removed and weighed on an electronic scale. Each gram of saliva was converted into 1 mm of saliva.	Total saliva:<br>G1 = 0.05 (±0.03)mL/min<br><br>G2 = 0.05 (±0.03)mL/min	Total saliva:<br>G1 = 0.28 (±0.15)mL/min<br><br>G2 = 0.17 (±0.10)mL/min	Increased flow after both treatments, with G1 being better than G2.
Studies in subjects with hyposalivation of unknown cause								
Blom et al. *<br><br>Switzerland<br><br>1992<br><br>RCT	Acupuncture	N G1 = 11 (Acupuncture)<br><br>Age G1 (mean) = 58 years<br><br>N G2 = 10 (Superficial acupuncturel)<br><br>Age G2 (mean) = 62 years<br><br>(In this case, only patient P, from G2).		G1 = 6 to 8 local and distal points and 2 to 4 auricular points, with insertions at depths between 0.5 and 2.0 cm and approximately 1 to 3 mm into the ear.<br><br>G2 = intraoperative needles dermal (superficial acupuncture).<br><br>G1 and G2 = 2x/week, 20 minutes, for 6 weeks. Interval of 7 to 10 days and protocol repetition for another 6 weeks.	Saliva collection unstimulated and stimulated by paraffin at two different times before starting treatment.<br><br>Samples were collected twice before the second series of acupuncture treatments.<br><br>New salivary assessments were made immediately after the completion of the acupuncture series; in 3 weeks; and 3, 6 and 12 months after the end of treatment.	USF = 0.04 mL/min<br><br>SSF = 0.38 mL/min	Immediately after:<br>USF = 0.01 mL/min<br><br>SSF = 0.46 mL/min<br><br>12 months later:<br>USF = 0.06 mL/min<br><br>SSF = 0.30 mL/min	G1 showed improved salivary flow rates during and after treatment. 4 out of 11 patients achieved normal salivary flow rates. The results persisted after 12 months.<br><br>G2 showed improvement in their salivary flow rates during treatment, but these changes disappeared after the conclusion of the protocol.<br><br>Patients using mechanical stimulants and salivary substitutes were excluded.
Studies in subjects with hypothyroidism								
Blom et al. *<br><br>Switzerland	Acupuncture	N G1 = 11 (Acupuncture)		G1 = 6 to 8 local and distal points and 2 to 4 auricular points, with insertions at	Saliva collection unstimulated and stimulated by paraffin at	USF = 0.01 mL/min	Immediately after:<br>USF= 0.12	G1 showed improved salivary flow rates during and after treatment. 4 out of

1992<br><br>RCT		Age G1 (mean) = 58 years<br><br>N G2 = 10 (Superficial acupuncturel)<br><br>Age G2 (mean) = 62 years<br><br>(In this case, only patient T, from G2).		depths between 0.5 and 2.0 cm and approximately 1 to 3 mm into the ear.<br><br>G2 = intraoperative needles dermal (superficial acupuncture).<br><br>G1 and G2 = 2x/week, 20 minutes, for 6 weeks. Interval of 7 to 10 days and protocol repetition for another 6 weeks.	two different times before starting treatment.<br><br>Samples were collected twice before the second series of acupuncture treatments.<br><br>New salivary assessments were made immediately after the completion of the acupuncture series; in 3 weeks; and 3, 6 and 12 months after the end of treatment.	SSF = 0.20 mL/min	mL/min<br><br>SSF = 0.26 mL/min<br><br>12 months later:<br>USF = 0.04 mL/min<br><br>SSF = 0.40 mL/min	11 patients achieved normal salivary flow rates. The results persisted after 12 months.<br><br>G2 showed improvement in their salivary flow rates during treatment, but these changes disappeared after the conclusion of the protocol.<br><br>Patients using mechanical stimulants and salivary substitutes were excluded.
Studies in individuals with heart disease								
Blom et al. *<br><br>Switzerland<br><br>1992<br><br>RCT	Acupuncture	N G1 = 11 (Acupuncture)<br><br>Age G1 (mean) = 58 years<br><br>N G2 = 10 (Superficial acupuncturel)<br><br>Age G2 (mean) = 62 years<br><br>(In this case, patients B and L, from G1).		G1 = 6 to 8 local and distal points and 2 to 4 auricular points, with insertions at depths between 0.5 and 2.0 cm and approximately 1 to 3 mm into the ear.<br><br>G2 = intraoperative needles dermal (superficial acupuncture).<br><br>G1 and G2 = 2x/week, 20 minutes, for 6 weeks. Interval of 7 to 10 days and protocol repetition for another 6 weeks.	Saliva collection unstimulated and stimulated by paraffin at two different times before starting treatment.<br><br>Samples were collected twice before the second series of acupuncture treatments.<br><br>New salivary assessments were made immediately after the completion of the acupuncture series; in 3 weeks; and 3, 6 and 12 months after the end of treatment.	USF = 0.02 mL/min<br><br>SSF = 0.27mL/min	Immediately after:<br>USF = 0.23 mL/min<br><br>SSF = 0.79 mL/min<br><br>12 months later:<br>USF = 0.18 mL/min<br><br>SSF = 0.72 mL/min	G1 showed improved salivary flow rates during and after treatment. 4 out of 11 patients achieved normal salivary flow rates. The results persisted after 12 months.<br><br>G2 showed improvement in their salivary flow rates during treatment, but these changes disappeared after the conclusion of the protocol.<br><br>Patients using mechanical stimulants and salivary substitutes were excluded.
Studies in subjects with hypertension								
Blom et al. *<br><br>Switzerland	Acupuncture	N G1 = 11 (Acupuncture)		G1 = 6 to 8 local and distal points and 2 to 4 auricular points, with insertions at	Saliva collection unstimulated and stimulated by paraffin at	USF = 0.05 mL/min	Immediately after:<br>USF = 0.06	G1 showed improved salivary flow rates during and after treatment. 4 out of

1992<br><br>RCT		Age G1 (mean) = 58 years<br><br>N G2 = 10 (Superficial acupuncturel)<br><br>Age G2 (mean) = 62 years<br><br>(In this case, patients O, Q, S of G2).		depths between 0.5 and 2.0 cm and approximately 1 to 3 mm into the ear.<br><br>G2 = intraoperative needles dermal (superficial acupuncture).<br><br>G1 and G2 = 2x/week, 20 minutes, for 6 weeks. Interval of 7 to 10 days and protocol repetition for another 6 weeks.	two different times before starting treatment.<br><br>Samples were collected twice before the second series of acupuncture treatments.<br><br>New salivary assessments were made immediately after the completion of the acupuncture series; in 3 weeks; and 3, 6 and 12 months after the end of treatment.	SSF = 0.39 mL/min	mL/min<br><br>SSF = 0.38 mL/min<br><br>12 months later:<br>USF = 0.08 mL/min<br><br>SSF = 0.36 mL/min	11 patients achieved normal salivary flow rates. The results persisted after 12 months.<br><br>G2 showed improvement in their salivary flow rates during treatment, but these changes disappeared after the conclusion of the protocol.<br><br>Patients using mechanical stimulants and salivary substitutes were excluded.
Blom et al. *<br><br>Switzerland<br><br>1992<br><br>RCT	Acupuncture	N G1 = 11 (Acupuncture)<br><br>Age G1 (mean) = 58 years<br><br>N G2 = 10 (Superficial acupuncturel)<br><br>Age G2 (mean) = 62 years<br><br>(In this case, only patient C, from G1).		G1 = 6 to 8 local and distal points and 2 to 4 auricular points, with insertions at depths between 0.5 and 2.0 cm and approximately 1 to 3 mm into the ear.<br><br>G2 = intraoperative needles dermal (superficial acupuncture).<br><br>G1 and G2 = 2x/week, 20 minutes, for 6 weeks. Interval of 7 to 10 days and protocol repetition for another 6 weeks.	Saliva collection unstimulated and stimulated by paraffin at two different times before starting treatment.<br><br>Samples were collected twice before the second series of acupuncture treatments.<br><br>New salivary assessments were made immediately after the completion of the acupuncture series; in 3 weeks; and 3, 6 and 12 months after the end of treatment.	USF = 0.04 mL/min<br><br>SSF = 0.40 mL/min	Immediately after:<br>USF = 0.16 mL/min<br><br>SSF= 0.76 mL/min<br><br>12 months later:<br>USF = 0.24 mL/min<br><br>SSF = 1.04 mL/min	G1 showed improved salivary flow rates during and after treatment. 4 out of 11 patients achieved normal salivary flow rates. The results persisted after 12 months.<br><br>G2 showed improvement in their salivary flow rates during treatment, but these changes disappeared after the conclusion of the protocol.<br><br>Patients using mechanical stimulants and salivary substitutes were excluded.
Studies in subjects with other rheumatic diseases								
Blom et al. *<br><br>Switzerland<br><br>1992<br><br>RCT	Acupuncture	N G1 = 11 (Acupuncture)<br><br>Age G1 (mean) = 58 years		G1 = 6 to 8 local and distal points and 2 to 4 auricular points, with insertions at depths between 0.5 and 2.0 cm and approximately 1 to 3 mm into the ear.	Saliva collection unstimulated and stimulated by paraffin at two different times before starting treatment.<br><br>Samples were collected	USF = 0.10 mL/min<br><br>SSF = 0.48 mL/min	Immediately after:<br>USF = 0.22 mL/min<br><br>SSF = 1.08 mL/min	G1 showed improved salivary flow rates during and after treatment. 4 out of 11 patients achieved normal salivary flow rates. The results persisted after 12 months.

		N G2 = 10 (Superficial acupuncturel)<br><br>Age G2 (mean) = 62 years<br><br>(In this case, only patient D, from G1).		G2 = intraoperative needles dermal (superficial acupuncture).<br><br>G1 and G2 = 2x/week, 20 minutes, for 6 weeks. Interval of 7 to 10 days and protocol repetition for another 6 weeks.	twice before the second series of acupuncture treatments.<br><br>New salivary assessments were made immediately after the completion of the acupuncture series; in 3 weeks; and 3, 6 and 12 months after the end of treatment.		12 months later:<br>USF = 0.30 mL/min<br><br>SSF = 1.10 mL/min	G2 showed improvement in their salivary flow rates during treatment, but these changes disappeared after the conclusion of the protocol.<br><br>Patients using mechanical stimulants and salivary substitutes were excluded.
Studies in healthy subjects without hyposalivation								
Aggarwal et al.<br><br>India<br><br>2015<br><br>SACT	TENS	N = 80<br><br>Age (mean) = Between 20 and 50 years		Electrodes bilaterally on the skin overlying the parotid gland region.<br><br>Frequency 100Hz and pulse duration 100-150µs.<br><br>After a 2-minute break, the TENS unit was activated and the amplitude was increased to a maximum tolerated level for 5 minutes.<br><br>Single application.	After the electrodes were positioned off, unstimulated saliva was collected ("forced spit" method) for 5 minutes.<br><br>After reaching the maximum intensity tolerated by the patient, the stimulated saliva was collected for 5 minutes.<br><br>The collections were performed before and after the application of TENS.	USF = 1.25 (±0.49)mL/min	SSF = 1.41 (±0.55)mL/min	Increase in mean salivary flow after TENS application (about 13%, 0.16 mL/min).<br><br>No control group to compare findings.
Bhasin et al.<br><br>India<br><br>2015<br><br>SACT	TENS	N = 100<br><br>Age = Between 20 and 69 years		Electrodes bilaterally on the skin overlying the parotid gland region.<br><br>The device used operates with a frequency of 0.1 to 500Hz. Amplitude was increased to a maximum tolerable level for the patient	Unstimulated saliva was collected ('forced spit' method) for 5 minutes.<br><br>After reaching the maximum intensity tolerated by the patient, the stimulated saliva was collected for 5 minutes.	Total saliva:<br><br>2.60 (± 0.39)mL/5 min	Saliva during stimulation: 3.60 (± 0.39)mL/5min<br><br>30 minutes later:<br>3.23 (±	There was no difference in different age groups (from 20 to 69 years old).<br><br>96% of patients responded positively to TENS therapy.<br><br>Significant increase after 30min and 24h.

				for 5 minutes.<br><br>Single application.	The collections were performed before, during the application (5mins), 30mins and 24h after the stimulation.		0.41)mL/5min<br><br>24 hours later: 2.69 (± 0.39) mL/5min	No control group to compare findings.
Chandra et al. *<br><br>India<br><br>2022<br><br>NRCT	TENS	N = 25 (health)<br><br>Age = Between 18 and 70 years		Electrodes bilaterally on the skin overlying the parotid gland region.<br><br>Frequency between 20-50 Hz, intensity according to the patient's tolerance.<br><br>2x/week every 15 days, for 1 month (4 times in total).	Unstimulated saliva ("forced spit" method) was collected for 5 minutes before TENS application.<br><br>After reaching the maximum tolerated intensity, stimulated saliva was collected for 5 minutes.<br><br>The difference between salivary flow after and before TENS application in the 4 sessions was calculated.	Mean difference in salivary flow in the 1st session:<br><br>G2 = 0.30(±0.14) mL/min	Mean difference in salivary flow in the 4th session:<br><br>G2 = 1.56(±0.28) mL/min	Increased salivary flow in G1 after treatment.<br><br>G2 showed a greater response than G1 after treatment.
Dawidson et al.<br><br>Sweden<br><br>1997<br><br>SACT	Acupuncture and electroacupuncture	N = 8<br><br>Age (mean) = 31.5 years		Acupuncture at 6 points bilaterally.<br><br>The same patients received low-frequency electrical stimulation (2 Hz) evoked with a Multiple Electronic Acupuntoscope. The needles at points St6 and Li4 were connected to the electropulse bilaterally, and the electrical current was adjusted between 2-4 mA. Electrical stimulation was applied for 20 minutes.<br><br>Single application.	Saliva was collected before the start of the study in different ways: unstimulated saliva, total paraffin-stimulated saliva, total saliva stimulated with 1% citric acid. The collection lasted 20 min under the described conditions.<br><br>The collected saliva was quantified by weight and 1 g was considered as a response to 1 mL.<br><br>Saliva was collected during the 20 minutes of treatment and, after removing the needles,	NA	Acupuncture:<br>Without stimulation = +28% (during) and +36% (after).<br><br>Paraffin stimulation = -11% (during) and -3% (after).<br><br>Stimulation with citric acid = -13% (during) and -0,1% (after).	Increased unstimulated salivary flow during and after manual acupuncture stimulation.<br><br>The mean increase in salivary flow rates was 28% during acupuncture treatment and 36% after needle removal.<br><br>Electrically stimulated acupuncture had no effect on unstimulated salivary flow.<br><br>Decreased saliva flow rates stimulated by chewing during electrically stimulated acupuncture. There were no

					saliva collection continued for another 20 minutes.<br><br>These three collections were repeated with electroacupuncture.<br><br>Differences (%) in salivary flow rates between baseline levels and during and after stimulation were given.		Electroacupuncture:<br><br>Without stimulation = +2% (during) and +16% (after).<br><br>Paraffin stimulation = -20% (during) and -14% (after).<br><br>Stimulation with citric acid = -13% (during) and -3% (after).	otherwise significant changes, but a trend towards decreased salivary flow rates.<br><br>No control group to compare findings.
Hargitai et al.<br><br>United States of America<br><br>2005<br><br>SACT	TENS	N = 22<br><br>Age(mean) = 35 years		Electrodes bilaterally on the skin overlying the parotid gland region.<br><br>The pulse rate was fixed at 50 Hz, the pulse duration was fixed at 250ms and the unit was in normal mode. Reach the maximum intensity tolerable by the patient. Application for 5 minutes.<br><br>Single application.	Unstimulated saliva was collected for 5 minutes (Carlson-Crittenden saliva collection cup was placed over Stensen's duct bilaterally).<br><br>After device activation, stimulated saliva was collected for 5 minutes.<br><br>Collection before and during device activity.	Total saliva = 0.02(±0.03) mL/min	Total saliva during stimulation = 0.05(±0.04) mL/min	15 of the 22 subjects showed an increase in salivary flow.<br><br>The difference between before and after is GIS.<br><br>The increase after stimulation was 1.86 to 8.75 times greater than the baseline value.<br><br>No control group to compare findings.
Nimma et al.<br><br>India<br><br>2012<br><br>SACT	TENS	N = 50<br><br>Age = Between 18 and 60 years		Electrodes bilaterally on the skin overlying the parotid gland region.<br><br>The pulse rate was fixed at 50 Hz and the amplitude was the maximum tolerated by the patient. Application for 5 minutes.<br><br>Method applied on 2	Unstimulated saliva was collected ('forced spit' method) for 5 minutes.<br><br>Stimulated saliva was collected after TENS application on the 1st and 2nd day.	Men: 2.02 (±0.65)mL/5min<br><br>Women: 2.01 (±0.59)mL/5min	1st day = 2.26 (±0.68)mL/5min<br><br>2nd day = 2.17 (±0.68)mL/min	Increased stimulated total saliva flow rate in 75% of subjects.<br><br>The amount of unstimulated and stimulated total saliva on day 2 showed a SIG increase in salivary flow rate after TENS.<br><br>The stimulated salivary flow

				consecutive days.				was higher on the 1st day compared to the 2nd day, with a SIG difference of 0.018mL/min (4%).<br><br>No control group to compare findings.
Pandey et al.<br><br>India<br><br>2019<br><br>SACT	TENS	N = 100<br><br>Age = Between 18 and 45 years.		Day 1: Citric acid stimulation for all subjects. Rinse with 5 mL of citric acid solution (25%) for 15s.<br><br>2nd day: Stimulation with TENS for all individuals. Electrodes bilaterally on the skin overlying the parotid gland region. The pulse rate was fixed at 50 Hz and the amplitude was the maximum tolerated by the patient. Application for 5 minutes.	Saliva samples were collected on 2 consecutive days.<br><br>Unstimulated saliva was collected before the stimulated saliva collection on both days ("forced spit" method) for 5 minutes.<br><br>On the 1st day, stimulated saliva was collected for 5 minutes after rinsing with a citric acid solution.<br><br>On the 2nd day, after the maximum intensity supported by the patients, the stimulated saliva was collected for 5 minutes.	1st day = 1.48(±0.28) mL/5min<br><br>2nd day = 1.49(±0.29) mL/5min	1st day (citric acid) = 5.52(±0.71) mL/5min<br><br>2nd day (TENS) = 4.33(±0.77) mL/5min	Increased salivary flow after stimulation with citric acid and TENS.<br><br>The increase in salivary flow was higher after stimulation with citric acid compared to stimulation with TENS.<br><br>No control group to compare findings.
Pattipati et al.<br><br>India<br><br>2013<br><br>SACT	TENS	N G1 = 30<br><br>Age G1 = 21 - 35 years.<br><br>N G2 = 30<br><br>Age G2 = 36 - 50 years.<br><br>N G3 = 30<br><br>Age G3 = Over 50 years.		Electrodes bilaterally on the skin overlying the parotid gland region.<br><br>50 Hz pulse rate, unit in normal mode.<br><br>Single application. No information regarding TENS application time.	Total saliva was collected immediately after electrostimulation and 1h after electrostimulation, asking the patient to spit into a graduated bottle.<br><br>It is not clear how long the saliva was collected and whether the flow is presented in ml/min.	NA	Immediately after:<br>G1 = 2.77(±0.26)<br><br>G2 = 2.41(±0.25)<br><br>G3 = 1.79(±0.16)<br><br>1 hour later:<br>G1= 2.33(±0.27)	Small variation in salivary flow rates between G1 and G2, between G2 and G3 groups and a moderate variation in salivary flow rates between G1 and G3 groups in both conditions.<br><br>No control (treatment) group to compare findings.

							G2= 1.97(±0.18)<br><br>G3= 1.49(±0.19)	
Ramesh et al.<br><br>India<br><br>2021<br><br>SACT	TENS	N = 130<br><br>Age = Between 20 and 49 years.		Electrodes bilaterally on the skin overlying the parotid gland region.<br><br>The pulse rate was fixed at 50 Hz and the amplitude was the maximum tolerated by the patient. Application for 5 minutes.<br><br>Single application.	Unstimulated saliva was collected ('forced spit' method) for 5 minutes.<br><br>After device activation, stimulated saliva was collected for 5 minutes.	Total saliva:<br><br>1.33(±0.05) mL/min	Total saliva:<br><br>1.44(±0.07) mL/min	117 patients showed an increase in salivary flow rate, while 10 showed no increase and 3 showed an unexpected decrease in salivary flow rate on TENS application.<br><br>No control group to compare findings.
Sing et al.<br><br>India<br><br>2015<br><br>SACT	TENS	N = 50<br><br>Age (mean) = 25.92 years		Electrodes bilaterally on the skin overlying the parotid gland region.<br><br>The amplitude was the maximum tolerated by the patient. Application for 5 minutes. Rate and pulse were not reported.<br><br>Single application.	Unstimulated saliva was collected ('forced spit' method) for 5 minutes.<br><br>After reaching the maximum intensity tolerated by the patient, the stimulated saliva was collected for 5 minutes.	Total saliva:<br><br>G1 = 0.35(±0.20) mL/min	Total saliva:<br><br>G1 = 0.49(±0.24) mL/min	43 out of 50 subjects showed an increase in salivary flow after stimulation.<br><br>No control group to compare findings.
Vilas et al.<br><br>India<br><br>2009<br><br>SACT	TENS	N = 100<br><br>Age = Between 18 and 75 years		Electrodes bilaterally on the skin overlying the parotid gland region.<br><br>The pulse rate was fixed at 50 Hz and the amplitude was the maximum tolerated by the patient. Application for 5 minutes.<br><br>Single application.	Unstimulated saliva was collected ('forced spit' method) for 5 minutes.<br><br>After reaching the maximum intensity tolerated by the patient, the stimulated saliva was collected for 5 minutes.	Total saliva:<br><br>0.36 (±0.16)mL/min	Total saliva:<br><br>0.42 (±0.19)mL/min	85% of subjects demonstrated increased total salivary flow after stimulation.<br><br>21% increase in salivary flow after TENS application.<br><br>No control group to compare findings.
